# Particle injection in three-dimensional relativistic magnetic reconnection

**DOI:** 10.1017/S0022377825101189

**Published:** 2026-02-19

**Authors:** Omar French, Gregory R. Werner, Dmitri A. Uzdensky

**Affiliations:** 1 Center for Integrated Plasma Studies, Department of Physics, University of Colorado, 390 UCB, Boulder, CO 80309-0390, USA; 2 Rudolf Peierls Centre for Theoretical Physics, University of Oxford, Oxford OX1 3NP, UK

**Keywords:** astrophysical plasmas, plasma simulation

## Abstract

Relativistic magnetic reconnection has been proposed as an important non-thermal particle acceleration (NTPA) mechanism that generates power-law spectra and high-energy emissions. Power-law particle spectra are in general characterised by three parameters: the power-law index, the high-energy cutoff and the low-energy cutoff (i.e. the injection energy). Particle injection into the non-thermal power law, despite also being a critical step in the NTPA chain, has received considerably less attention than the subsequent acceleration to high energies. Open questions on particle injection that are important for both physical understanding and astronomical observations include how the upstream magnetisation 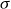 influences the injection energy and the contributions of the known injection mechanisms (i.e. direct acceleration by the reconnection electric field, Fermi kicks and pickup acceleration) to the injected particle population. Using fully kinetic particle-in-cell simulations, we uncover these relationships by systematically measuring the injection energy and calculating the contributions of each acceleration mechanism to the total injected particle population. We also present a theoretical model to explain these results. Additionally, we compare two- and three-dimensional simulations to assess the impact of the flux-rope kink and drift-kink instability on particle injection. We conclude with comparisons with previous work and outlook for future work.

## 1. Introduction

The origin of high-energy emissions in the Universe is often a non-thermal power-law spectrum of relativistic particles. Therefore, to explain high-energy emissions, it is necessary to understand how power-law distributions of non-thermal particles are populated and sustained, with the former being the concern of injection studies and the latter being the concern of studies about non-thermal particle acceleration to high energies.

The question of how a power-law distribution of particles is populated may be decomposed into two questions: (i) Under what conditions are particles injected, i.e. eligible to participate in a continual, power-law-forming acceleration process? (ii) What physical mechanisms are responsible for injection?

Regarding the first question, the injection criterion has typically been expressed since Fermi (1949) as an energy threshold 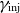 that a particle must surpass, so that particles with energy 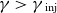 are ‘injected’ and can experience further Fermi acceleration to even higher energies, whereas those with 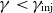 cannot. Physically, this may be owed to the relativistic gyroradius of particles scaling linearly with 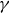 , which facilitates acceleration via stochastic scattering off of turbulent fluctuations (Lemoine & Malkov 2020). In this study, we also presuppose that 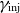 exists and define it as the low-energy boundary of the non-thermal power-law segment of the downstream particle distribution function. Thus, the first question is recast as a problem of relating 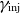 to the system parameters and explaining the connection.

Accordingly, the second question is also recast: it is to understand what ‘injection mechanisms’ – mechanisms that accelerate particles from a thermal upstream to or beyond the injection energy 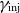 that gates the non-thermal power-law spectrum – are active, how much work they do to each particle and upon how many particles they act.

These questions of particle injection have been largely unresolved across several processes that are thought to power high-energy emissions in relativistic astrophysical environments, such as jets from active galactic nuclei, pulsar wind nebulae, neutron star magnetospheres and accreting black hole coronae. These processes include relativistic turbulence (Chandran 2000; Zhdankin *et al*. 2017; Comisso & Sironi 2018, 2019; Zhdankin *et al*. 2018, 2019; Lemoine 2019; Wong *et al*. 2020; Demidem, Lemoine & Casse 2020; Lemoine & Malkov 2020; Guo *et al*. 2025; Mehlhaff, Zhou & Zhdankin 2025), collisionless relativistic shocks (Blandford & Ostriker 1978; Blandford & Eichler 1987; Spitkovsky 2008; Sironi & Spitkovsky 2011; Caprioli & Spitkovsky 2014; Parsons, Spitkovsky & Vanthieghem 2024) and relativistic magnetic reconnection (Bulanov & Sasorov 1976; Blackman & Field 1994; Zenitani & Hoshino 2001; Jaroschek, Lesch & Treumann 2004; Giannios, Uzdensky & Begelman 2010; Cerutti *et al*. 2013; Sironi & Spitkovsky 2014; Melzani *et al*. 2014; Guo *et al*. 2014, 2015, 2016, 2019; Nalewajko *et al*. 2015; Werner *et al*. 2016; Rowan, Sironi & Narayan 2017; Werner & Uzdensky 2017; Petropoulou & Sironi 2018; Werner *et al*. 2018; Schoeffler *et al*. 2019; Kilian *et al*. 2020; Mehlhaff *et al*. 2020; Hakobyan *et al*. 2021; Werner & Uzdensky 2021; Sironi 2022; Uzdensky 2022; French *et al*. 2023; Li *et al*. 2023; Zhang *et al*. 2023; Gupta, Sridhar & Sironi 2025) (see Guo *et al*. 2024; Sironi, Uzdensky & Giannios 2025, for recent reviews).

In the context of relativistic magnetic reconnection, several injection and high-energy acceleration mechanisms have been studied, both analytically and numerically via fully kinetic particle-in-cell (PIC) simulations: ‘direct’ acceleration by the parallel electric field with a finite guide magnetic field (i.e. a finite non-reversing, out-of-plane component of the magnetic field) near X-points (Larrabee, Lovelace & Romanova 2003; Zenitani & Hoshino 2005, 2008; Cerutti *et al*. 2013, 2014; Ball, Sironi & Özel 2019; Sironi 2022; Totorica *et al*. 2023; Gupta *et al*. 2025), Speiser orbits in the case of zero guide field (Speiser 1965; Hoshino *et al*. 2001; Zenitani & Hoshino 2001; Uzdensky, Cerutti & Begelman 2011; Cerutti *et al*. 2012, 2013, 2014; Nalewajko *et al*. 2015; Uzdensky 2022), Fermi acceleration (Fermi 1949; Drake *et al*. 2006; Giannios *et al*. 2010; Guo *et al*. 2014, 2015; Dahlin, Drake & Swisdak 2014; Zhang *et al*. 2021; French *et al*. 2023; Zhang *et al*. 2024), parallel electric field acceleration in the exhaust region (Egedal & Daughton 2013; Zhang, Drake & Swisdak 2019) and acceleration by the pickup process in the exhaust region (Drake *et al*. 2009; Sironi & Beloborodov 2020; Chernoglazov, Hakobyan & Philippov 2023; French *et al*. 2023). Previous work on particle injection in magnetic reconnection has included studies in the transrelativistic regime for a proton–electron plasma with a weak guide field (Ball *et al*. 2019; Kilian *et al*. 2020) and relativistic pair plasmas for various guide-field strengths (Sironi 2022; French *et al*. 2023; Guo *et al*. 2023; Totorica *et al*. 2023) and upstream magnetisations (Sironi 2022; Guo *et al*. 2023; Totorica *et al*. 2023; Gupta *et al*. 2025).

However, these studies have not elucidated several important aspects of injection, such as how the injection stage in relativistic magnetic reconnection is influenced by the upstream magnetisation and three-dimensional (3-D) effects. Addressing this key question is the subject of this paper. We achieve this using analytical theory and fully kinetic 2-D and 3-D PIC simulations of relativistic magnetic reconnection in non-radiative collisionless pair plasmas. To uncover spectral quantities, most importantly the injection energy 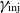 , we run a spectral fitting procedure that is improved from our previous work (French *et al*. 2023). To uncover the relative contributions of different injection mechanisms, we employ a methodology similar to French *et al*. (2023), wherein we consider several mechanisms, namely, parallel electric fields near X-points, Fermi kicks by the motional electric field and the pickup process.

Throughout this paper, we use the units 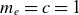 . That is, we normalise velocities to the speed of light 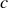 , momenta to 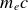 and energies to the electron rest energy 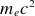 . Furthermore, we denote 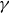 as the energy of a single particle, 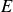 as the sum or integral of energies of multiple particles, and 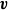 as the particle 3-velocity in the simulation frame. Primed vector quantities, such as the velocity 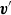 or the momentum 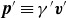 , denote a Lorentz boost to the 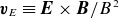 drift-velocity frame, where 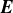 and 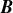 respectively denote the electric and magnetic field vectors. This is done to eliminate the context of bulk plasma motions. Primed scalar quantities denote those whose vector inputs are primed, e.g. 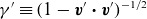 . Subscripts 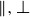 indicate components relative to the local magnetic field in the laboratory frame ( 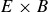 drift-velocity frame) if the quantity is not primed (primed). Lastly, we reference four dimensionless quantities used throughout this paper: the cold upstream magnetisation
(1.1)

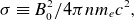

corresponding to the average magnetic energy per particle rest mass energy (where 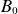 is the ambient upstream magnetic field and 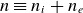 is the total upstream plasma density), the hot upstream magnetisation
(1.2)

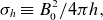

where 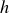 is the relativistic plasma enthalpy density, the ambient upstream temperature normalised by particle rest mass energy
(1.3)

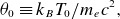

and the normalised guide-field strength
(1.4)

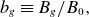

where 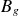 is the `guide magnetic field,’ a uniform, non-reversing component of the magnetic field that orients out of plane.

The rest of this paper is organised as follows. Section 2 presents our theoretical picture of particle injection. Section 3 describes the set-up for the simulations. Section 4 contains the analysis and results from each simulation. Section 5 discusses comparisons with previous work and outlooks for future work. Section 6 presents our main conclusions.

## 2. Theoretical picture of particle injection

In concordance with our overview in § 1, our picture of particle injection in relativistic magnetic reconnection has two components. The first is an analytical model for the injection energy 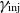 (§ 2.1). The second is a detailed description of each mechanism that injects particles (§ 2.2).

### 2.1. Injection criterion

Suppose the criterion for a particle to be ‘injected’, regardless of the mechanism of injection, is that its gyroradius 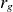 exceeds the reconnection layer half-thickness at the *X*-point 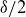 , i.e. 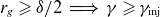 (Speiser 1965; Giannios *et al*. 2010). This criterion ensures that a gyrating particle centred on the X-point spends some fraction of each orbit outside the layer, which causes it to adopt a meandering Speiser trajectory. It follows that a newly injected particle will satisfy
(2.1)

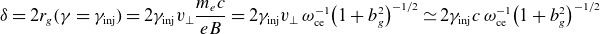



(2.2)

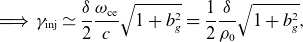

where 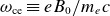 is the nominal electron gyrofrequency defined with the upstream magnetic field 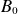 and 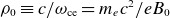 is the corresponding nominal relativistic gyroradius. In (2.1) we have ignored pitch angle corrections and assumed 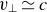 .

Our next task is to estimate the layer thickness 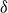 at the X-point. We shall take 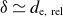 , where 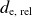 is the relativistic collisionless electron skin depth inside the layer, and assume that the electron density in the layer is comparable to the ambient upstream electron density 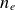 . Additionally, we assume that the velocity distribution is isotropic in the layer. Then,
(2.3)

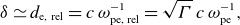

where 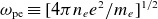 is the corresponding electron plasma frequency and 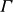 is the mean Lorentz factor of electrons in the layer.

Substituting (2.3) into (2.2), we obtain
(2.4)

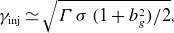

where for a pair plasma 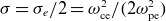 is the upstream magnetisation.

The remaining task is to model 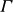 , which can be represented as the sum of two contributions
(2.5)

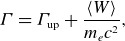

where 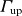 is the average Lorentz factor of particles arriving from the upstream and 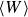 is the average work done to particles while they are in the layer. If the upstream particle distribution is thermal, then 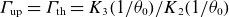 , where 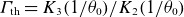 is the ‘thermal Lorentz factor’, i.e. the average energy of electrons in a Maxwell–Jüttner distribution of upstream temperature 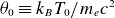 .

Meanwhile, 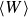 can generally be written 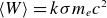 , where one expects the dimensionless coefficient 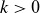 to be a constant independent of 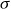 . We may constrain 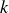 as follows. First, 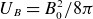 implies that the available magnetic energy per particle is 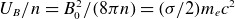 . Second, assuming that the efficiency of energy conversion is 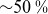 , the average dissipated magnetic energy per particle is 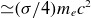 . Third, since the particles are energised partially as they enter the current sheet and partially as they exit the current sheet (e.g. by magnetic tension release in the outwards-accelerating plasma), we may assume that the average dissipated magnetic energy per particle that exists in the current sheet is 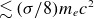 . This gives a constraint of 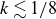 . We stress that, while the exact value of 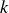 is uncertain, the above argument allows us to treat 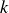 as a small parameter. Accordingly, we shall retain 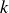 in the rest of this subsection.

We can now write (2.5) as
(2.6)

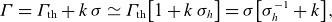

where we approximate 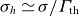 . We see that there are two distinct relativistic ( 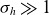 ) regimes based on which of the two terms in (2.6) dominates, i.e. how 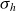 compares with 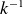 :

- **Thermally dominated regime** 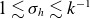 . In this moderate-magnetisation case (covering 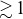 decade in 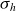 ), the average particle energy in layer is 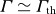 , i.e. inherited from the thermal upstream and not controlled by 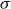 or reconnection. In this regime, the upstream plasma conditions fully govern the layer thickness and partially govern the injection energy 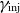 . In particular, the injection energy according to (2.4) becomes
(2.7)

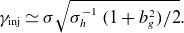

In particular,
(2.8)

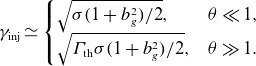

- **Magnetically dominated regime** 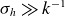 **.** In this case, the average particle energy in the layer 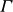 is dominated by 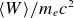 and upstream contributions can be ignored. Hence (2.4) becomes
(2.9)

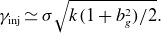

There are several implications for the resulting dynamic range of the power-law spectrum. Assuming that 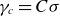 (Werner *et al*. 2016), one expects
(2.10)

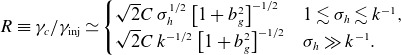




In this model, the explicit guide-field dependence of 
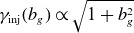
 is owed to stronger magnetic fields decreasing the particle gyroradius, resulting in a greater energy necessary to satisfy 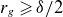 . There could also be an implicit guide-field dependence if the layer thickness depends on guide-field strength. This is plausible because a strong guide field can prolong the duration over which particles remain in the X-point, causing 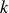 to increase. Nevertheless, as we shall find in § 5.1, the scaling 
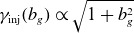
 is in rough agreement with French *et al*. (2023).

### 2.2. Injection mechanisms

**Figure 1.**
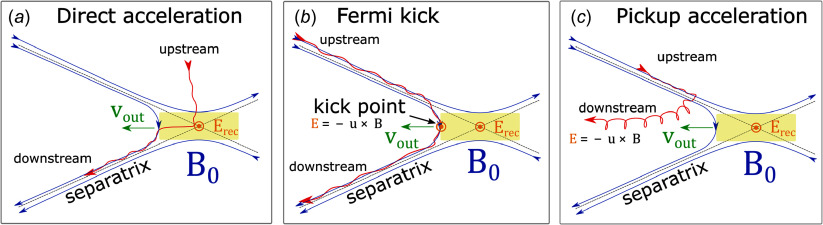
Cartoons of several particle injection mechanisms, adapted from French *et al*. (2023). In each panel, 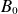 is the reconnecting magnetic field, 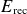 is the reconnection electric field and 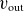 is the reconnection outflow speed. (*a*) Injection by direct acceleration from the reconnection electric field near an X-point. (*b*) Injection by a Fermi `kick.’ (*c*) Injection by the pickup process, wherein 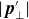 suddenly increases upon crossing the separatrix and subsequent entry into the downstream region.

In our first paper (French *et al*. 2023), we stipulated that particles can be injected only as they transition from upstream to downstream, i.e. as they cross or interact with the magnetic separatrix between these two regions. Applying this assumption in the context of a reconnection layer has led to the identification of the following three injection mechanisms (French *et al*. 2023), illustrated in figure 1.

- Direct acceleration: incoming particles are accelerated directly by the reconnection electric field 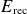 in microscopic diffusion regions around magnetic X-points (figure 1 *a*). When immersed in 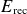 , charged particles gain energy at a rate of 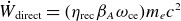 , where 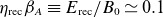 (when the guide magnetic field is weak) is the reconnection rate normalised by the speed of light, where 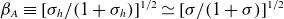 is the dimensionless upstream Alfvén speed (provided the upstream plasma is relativistically cold, i.e. 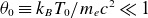 ) and 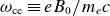 . Hence
(2.11)

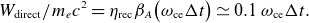

If a guide field 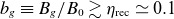 is present, then 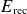 is well approximated by the parallel electric field 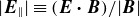 at the X-point where the in-plane magnetic field vanishes. In this case, particles gain significant parallel momentum 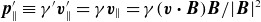 .
- Fermi kick: the relaxation of freshly reconnected magnetic field-line tension gives rise to a Fermi acceleration process wherein the local curvature drift velocity 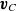 of particles is aligned with the motional electric field of ideal magnetohydrodynamics (MHD) 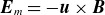 associated with the rapid advection of the newly reconnected magnetic field lines (Drake *et al*. 2006; Dahlin *et al*. 2014; Guo *et al*. 2014; Li *et al*. 2018; Kilian *et al*. 2020; Zhang *et al*. 2021; French *et al*. 2023; Majeski & Ji 2023; Zhang *et al*. 2024). This mechanism is illustrated in figure 1(*b*).
  Consequently, an incoming particle experiences one Fermi reflection (or ‘kick’, i.e. one half-cycle of a continual Fermi process) that flips its direction in the 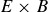 drift frame. If the Fermi kick is treated as a 1-D collision, then the net energy gain is obtained by considering the particle’s initial velocity in the outflow (i.e. 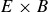 -drift) frame projected onto the outflow direction, 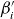 , negating it (i.e. 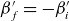 ), and boosting back to the simulation frame. Assuming that the outflow speed is the in-plane Alfvén speed, the result is a velocity gain of
(2.12)

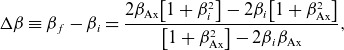

where 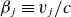 for any subscript 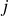 and 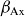 is the in-plane Alfvén speed 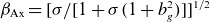 . As 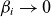 (i.e. for particles with initial velocity nearly perpendicular to the outflow), the resulting energy gain becomes
(2.13)

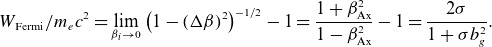

Hence a strong guide field (i.e. 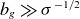 ) significantly damps Fermi kicks, resulting in an energisation of 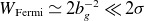 . Conversely, a weak guide field ( 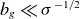 ) allows efficient energisation with 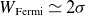 .
- Pickup acceleration: an incoming upstream particle crosses the reconnection separatrix suddenly and thus (i) the particle’s magnetic moment in the outflow frame 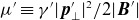 is no longer constant, resulting in a greater perpendicular momentum 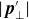 and (ii) the particle becomes immersed in a reconnection outflow with bulk ( 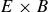 -drift) velocity of 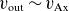 . The ideal-MHD (i.e. motional) electric field 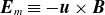 subsequently accelerates the particle until its perpendicular guiding-centre velocity matches the 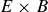 -drift velocity of the outflow (figure 1 *c*) (Drake *et al*. 2009; Chernoglazov *et al*. 2023; French *et al*. 2023). Thus the total work done by this pickup mechanism on a particle of initial energy 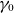 is
(2.14)

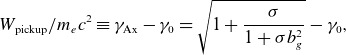

where 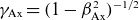 .

Several strategies have been employed in the literature to disentangle the energetic contribution of each mechanism to particle energisation. One common approach utilises the motional (i.e. ideal) electric field 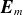 and the non-ideal electric field 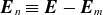 by approximating 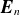 as the electric field component parallel to the local magnetic field, 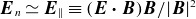 , and 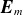 as the perpendicular component, 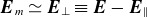 ; one then compares the works 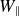 and 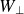 done by these electric-field components over a statistically large ensemble of tracer particles (Comisso & Sironi 2019; Guo *et al*. 2019; Kilian *et al*. 2020; French *et al*. 2023). Since the value of the normalised guide-field strength adopted in our present study, 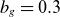 , exceeds the dimensionless reconnection rate 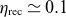 (Guo *et al*. 2015; Liu *et al*. 2015, 2017; Werner *et al*. 2018; Liu *et al*. 2020; Goodbred & Liu 2022; Zhang *et al*. 2023), which quantifies the typical strength of the downstream reconnected in-plane magnetic field relative to the upstream reconnecting magnetic field, we shall proceed with this approximation.

Thus, in this work we compute the relative contributions of each mechanism to the total injected particle population (i.e. ‘injection shares’) by subjecting each tracer particle in the ensemble to the following scheme (French *et al*. 2023). Upon reaching 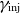 (i.e. when the particle is ‘injected’), the particle is endowed with its ‘injection time’ 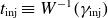 , where 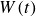 is the total time-evolved work done to the particle and 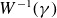 is its inverse function. Then the particle is assigned to a single mechanism according to whichever of the following conditions is true at 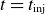 :
(2.15)

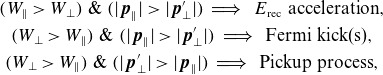

with the remaining possibility categorised as `other.’ Each 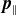 is left unprimed because boosting to the 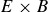 -drift frame makes no change to momenta parallel to the local magnetic field.

## 3. Simulation set-up

To study particle injection and acceleration by relativistic magnetic reconnection, we perform an array of fully kinetic simulations of a relativistic collisionless pair plasma using the Zeltron code, which solves the relativistic Vlasov–Maxwell equations (Cerutti *et al*. 2013) in 2-D and 3-D rectangular domains. All of our simulations are initialised with a force-free current sheet (CS) with the associated initial magnetic field
(3.1)

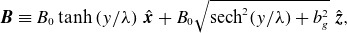

where 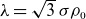 is the half-thickness of the initial CS and 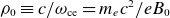 is the nominal relativistic gyroradius.

The pair mass ratio is 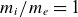 . The initial plasma density 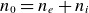 is uniform and represented by 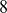 ( 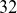 ) positron–electron pairs per computational grid cell in three dimensions (two dimensions), as justified in Appendix A. Currents are normalised by 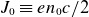 . The initial plasma is thermal with a uniform temperature 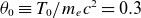 . The guide-field strength is set to 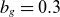 .

To examine the effects of magnetisation and dimensionality, our simulations vary these quantities independently. In particular, we scan the cold upstream magnetisation parameter over eight values: 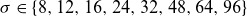 , where the latter three values are exclusive to two dimensions due to limited computational resources. Since 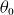 is sub-relativistic, these 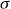 values are close to their corresponding ‘hot’ upstream magnetisations 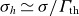 . To examine the effect of dimensionality we compare 2-D and 3-D simulations that are otherwise identical.

In this study, we characterise system sizes by the dimensionless measure 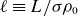 , where 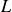 is the system size. The spatial domains of our simulations are rectilinear boxes with 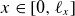 and 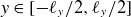 , and in three dimensions also with 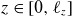 . The aspect ratio is fixed to 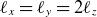 ( 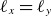 ) in three dimensions (two dimensions). To resolve kinetic scales, the spatial resolution is set to 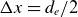 , as justified in Appendix A (where 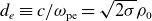 is the relativistic collisionless electron skin depth). The temporal resolution is set to 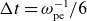 . In the 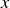 and 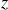 -directions, periodic boundary conditions are set for fields and particles, whereas in the 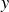 -direction conducting boundaries are set for fields and reflecting boundaries are set for particles. Instead of adding a small perturbation to trigger magnetic reconnection, we instead wait for the CS to start reconnecting spontaneously.^1^

The simulation domain size is fixed to 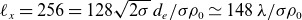 , to yield results that are converged in large-system-size limit (informed by our previous work; cf. French *et al*. (2023)). Correspondingly, we use 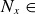 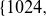 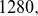 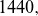 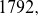 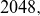 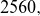 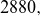 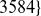 computational grid cells in the 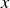 -direction across the 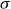 scan, where accordingly the latter three values are exclusive to two dimensions. The running time varies for each simulation, but is set to allow enough time for the plasma to (*a*) reconnect and (*b*) evolve for 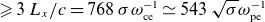 after reconnection onsets. This duration is sufficient for evolving quantities to saturate or otherwise reach steady-state evolution.

In this paper, we wish to investigate particle spectra in the ‘downstream,’ i.e. the region between the two separatrices. To isolate the downstream region, we apply the particle-mixing approach (Daughton *et al*. 2014) as follows. Each computational grid cell is assigned a mixing fraction 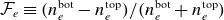 , where 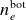 and 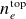 are the number densities of electrons that start at 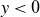 and 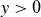 , respectively. Then, we consider cells which satisfy 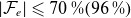 to be sufficiently well mixed to be regarded as ‘downstream’ in three dimensions (two dimensions) and the remaining cells are considered ‘upstream’ in three dimensions (two dimensions).^2^

When evaluating the contributions of each injection mechanism (see § 4.4), we randomly select 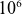 particles at the beginning of each simulation and track relevant physical quantities associated with them at each time step, including velocities and electric and magnetic fields. With this information, we study the acceleration mechanisms of particles statistically (Guo *et al*. 2016, 2019; Li *et al*., 2019*a*, *b*; Kilian *et al*. 2020; French *et al*. 2023). We exclude the initial current-carrying (i.e. drifting) particles from our tracer particle analysis.

## 4. Results

**Figure 2.**
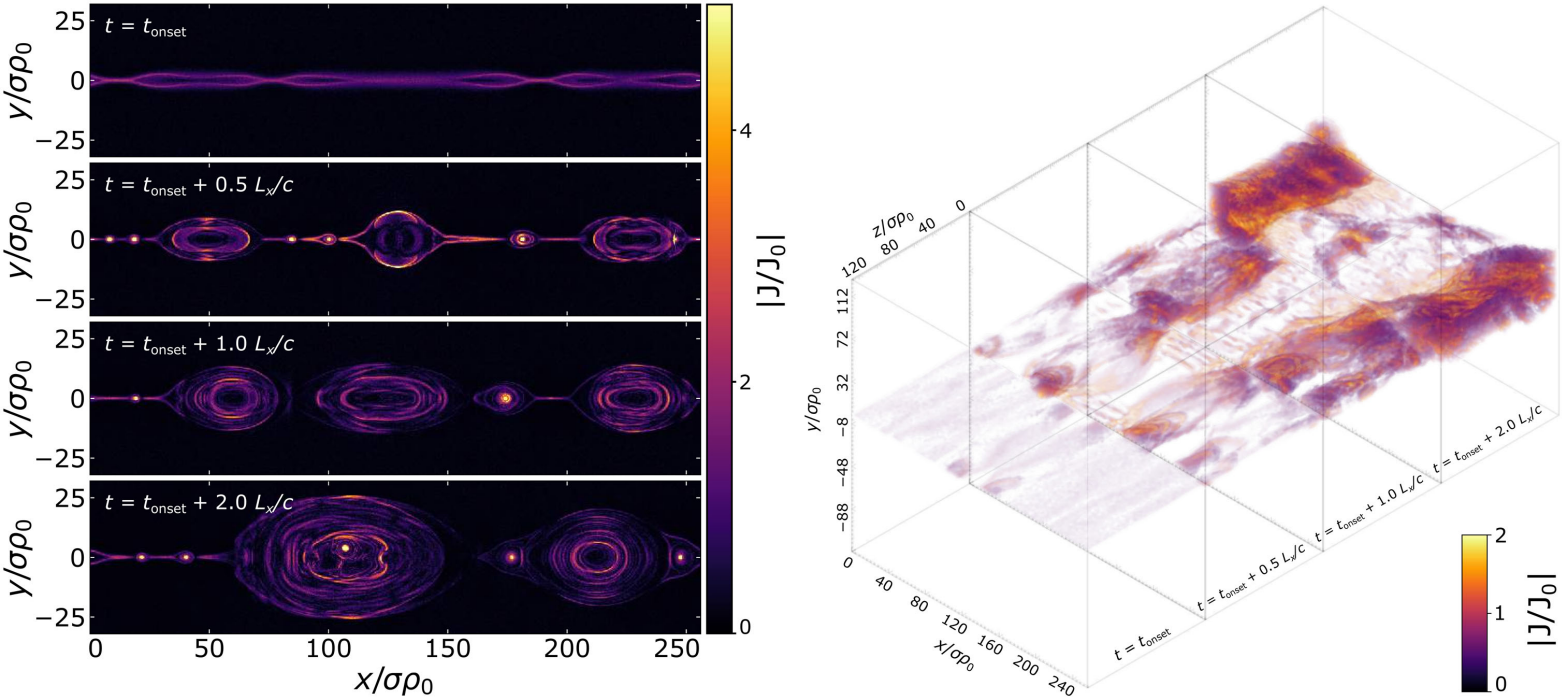
Absolute current density 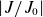 at different times after the time of reconnection onset 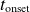 . Panels on the left display a 2-D simulation ( 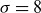 ) and the panels on the right display an otherwise identical 3-D simulation.

First, to illustrate the reconnection process, we show several snapshots of absolute current density 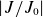 (where 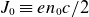 and 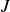 is the total current density) of two simulations with 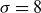 (figure 2). The left-hand panels display a 2-D simulation and the right-hand panels display an otherwise identical 3-D simulation. To make the downstream visible in three dimensions, we apply a linear ramp of increasing opacity for 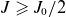 (opacity = 50 % at 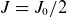 and opacity = 100 % at 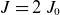 ) and set opacity = 0 for 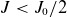 .

The earliest time displayed is the reconnection onset time 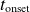 , defined as when the magnetic energy drops to 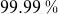 of its initial value. This threshold is sufficient for 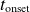 to demarcate the very first X-point collapse. Henceforth we will shift to this time for time-dependent results.

Accordingly, initial X-point collapse in two dimensions is shown to coincide with 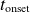 in the top-left panel of figure 2. Soon thereafter, plasmoids are advected downstream ( 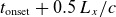 ), merge with other plasmoids ( 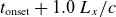 ) and retain their structural integrity throughout ( 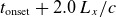 ). By contrast, in three dimensions the flux-rope kink instability dismantles plasmoids, allowing particles to escape from them (Dahlin *et al*. 2014; Werner & Uzdensky 2021; Zhang *et al*. 2021).

### 4.1. Reconnection rate

We define the dimensionless time-dependent reconnection rate 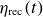 as the rate at which the unreconnected (i.e. upstream) magnetic flux 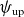 decays with time, normalised by 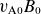



(4.1)

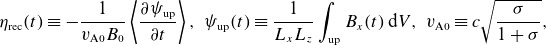

where 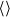 is the time averaging performed every 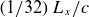 and the upstream region is defined by cells with a mixing fraction 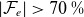 ( 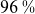 ) in three dimensions (two dimensions). The peak reconnection rate is defined simply as 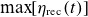 .

**Figure 3.**
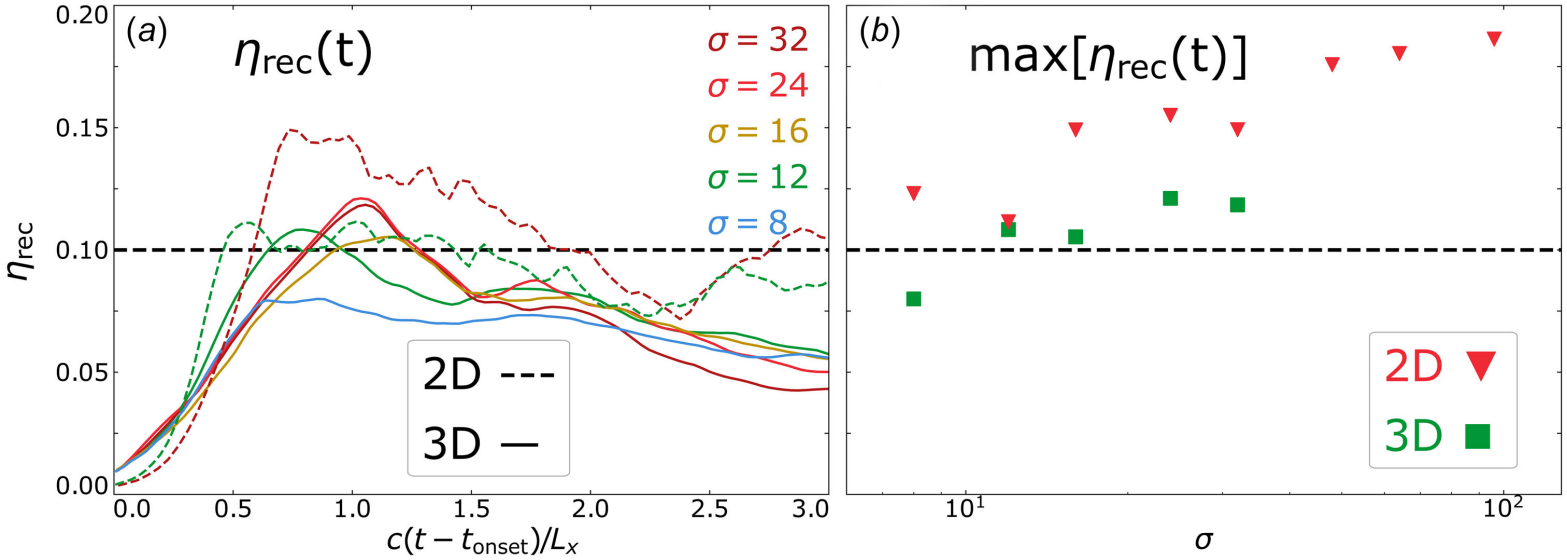
Reconnection rates for various 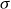 . (*a*) Time-dependent reconnection rates of 3-D (solid) and a few 2-D (dashed) simulations. (*b*) Peak reconnection rates, with green squares representing three dimensions and red triangles representing two dimensions.

In accordance with (4.1), we compute the time-dependent and peak reconnection rates for several values of 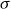 , as shown in figure 3. We find that the reconnection rate in three dimensions is consistently somewhat lower than each 2-D counterpart for every tested value of 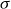 , and adheres quite closely to the canonical value of 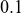 . As for 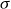 -dependence, the peak reconnection rate 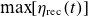 increases gradually with increasing 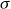 , from 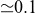 to 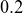 in two dimensions as 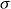 varies from 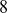 to 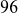 and from 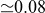 to 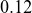 in three dimensions as 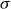 varies from 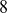 to 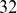 .

### 4.2. Injection energies

**Figure 4.**
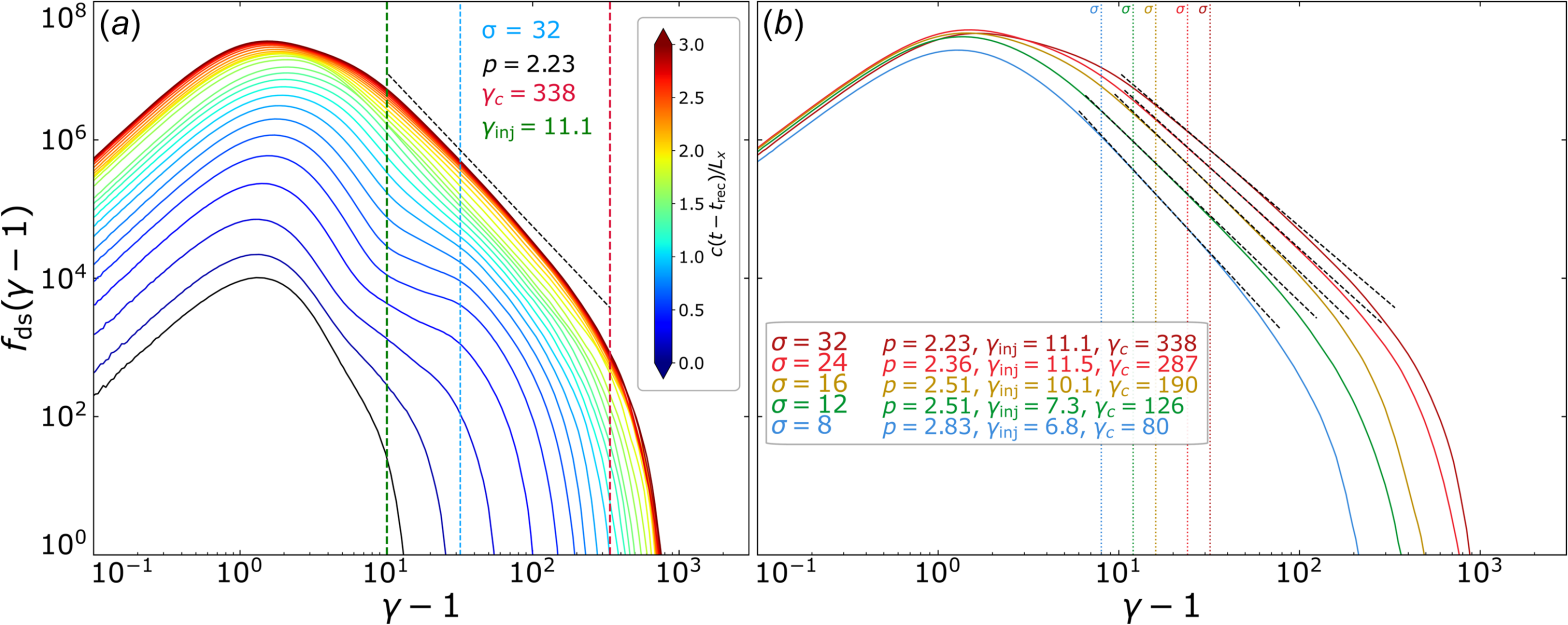
Downstream particle spectra from 3-D simulations. (*a*) Evolving downstream particle spectrum from a 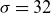 3-D simulation fitted at the final time step. The vertical dashed green line indicates the measured injection energy 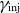 , the vertical dashed red line the measured cutoff energy 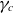 , and the dashed black line is 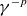 with measured power-law index 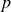 . Solid colour lines show particle spectra taken every 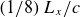 , from 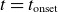 to 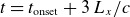 . (*b*) Downstream particle spectra of 3-D simulations at times 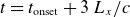 for various initial upstream magnetisations 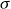 . Dashed black lines show 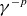 for 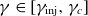 using the measured values of 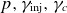 and dotted vertical lines are coloured and positioned at 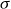 values.

To fit particle spectra, we perform a spectral fitting procedure described in Appendix B. To illustrate the process for acquiring the characteristic spectral parameters, we show a time-evolved downstream particle spectrum 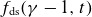 in figure 4(*a*). The downstream particle population is continuously fed by upstream particles crossing over the separatrix.

Measuring the characteristic parameters 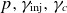 helps glean their dependencies on system parameters. In figure 4(*b*), we show several downstream particle spectra of 3-D simulations at the time 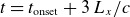 for various values of 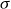 , as well as power-law fits that extend from the injection energy to the cutoff energy. This figure highlights the precision of measurement that is afforded by implementing the spectral fitting procedure described in Appendix B.

**Figure 5.**
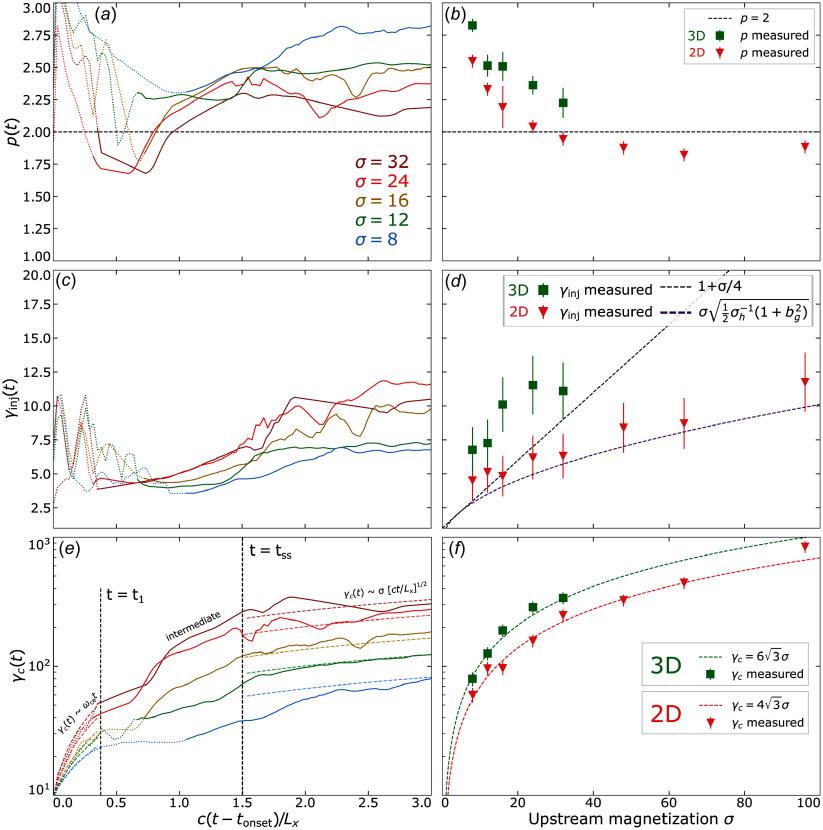
Spectral parameters measured via fitting procedure (described in Appendix B) for various 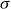 at each time step for 3-D simulations (*a*, *c*, *e*) and at 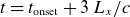 for all simulations (*b*, *d*, *f*). Dotted coloured lines in (*a*, *c*, *e*) indicate time steps where the power-law extent is short, i.e. 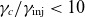 . In (*b*, *d*, *f*), red triangles are 2-D runs and green squares are 3-D runs. (*a*, *b*) Power-law indices 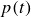 and 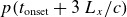 . (*c*, *d*): Injection energies 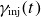 and 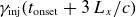 . The dashed black line in (*d*) shows linear scaling, assuming 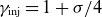 and the dashed purple line shows 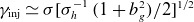 (i.e. (2.7)), derived in § 2.1. (*e*, *f*) High-energy cutoffs 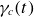 and 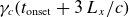 . The semi-transparent dashed coloured lines in (*e*) show the fit from (4.2) and the green (red) dashed line in (*f*) shows it evaluated at 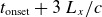 , i.e. 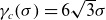 in three dimensions ( 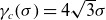 in two dimensions).

In the left-hand panels of figure 5 we show the evolved characteristic parameters of the power-law particle spectra ( 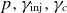 ) for various values of 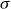 represented by curves of different colour. Data points are obtained every 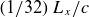 using the fitting procedure described in Appendix B and we smooth the data with a moving time average of window size 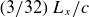 , i.e. where each value is replaced by the average of itself, its predecessor and its successor. In the right-hand panels of figure 5 we plot each measured parameter evaluated at 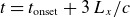 against 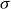 .

We find that power-law indices 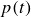 (top row of figure 5) rapidly harden during the transient phase ( 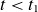 ), followed by a longer period whereupon they gradually soften, achieving stability 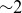 light-crossing times after 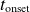 (panel a). Power-law spectra harden with increasing 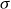 and are harder in two dimensions than in three dimensions by 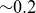 – 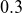 for any given value of 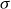 (panel b).

The injection energy 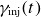 (middle row of figure 5) quickly adopts an initial (i.e. at 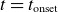 ) value of 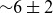 without a clear dependence on 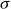 . While erratic, the injection energy remains roughly within this range during the transient reconnection phase ( 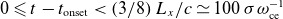 ). Once steady state is reached (e.g. after 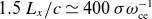 in three dimensions), the injection energy stabilises roughly on the same time scale with which 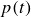 stabilises (panel c). At 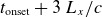 , the measured injection energies in two dimensions adhere reasonably closely to our model for the injection energy in the thermally dominated case 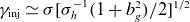 (cf. (2.7)) (dashed purple line) and is greatly exceeded by the often-assumed linear relation of 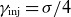 (indicated by the dashed black line).

In three dimensions, injection energies are consistently greater by a factor of 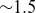 than in two dimensions, possibly owed to greater CS thicknesses at disruption in three dimensions vs two dimensions (cf. § 2.1). However, the large errors and the limited covered range of 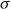 makes a definite scaling trend difficult to decipher. We compare these results with previous work in § 5.1.

Although not the primary concern of this study, we measure the high-energy cutoff 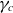 to grow rapidly during the transient reconnection phase 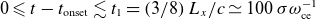 , in both two and three dimensions. By assuming 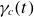 to scale with 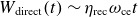 , we apply the fit 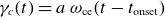 , where 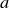 is a fitting parameter. Performing this fit in three dimensions yields the coefficients 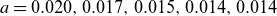 for 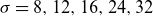 . The sensitivity of 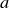 to 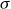 declines as the 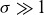 regime is entered. These fits are plotted on figure 5(*e*).

The steady-state phase ( 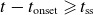 , where 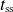 is the steady-state time) is characterised by the time interval when the simulation domain is populated with multiple plasmoids and the cutoff grows steadily. In three dimensions, the transition from the transient to the steady-state phase takes 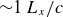 to complete for 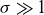 , with longer transition times at moderate values of 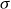 (cf. 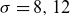 ). Thus 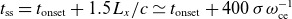 . During the steady-state phase ( 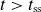 ), 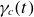 is fitted reasonably well by 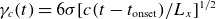 for every value of 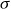 in three dimensions (panel e). Thus the complete cutoff energy fit in three dimensions is (where 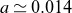 in the 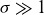 limit)
(4.2)

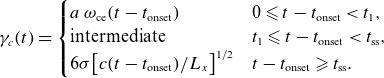




By contrast, in two dimensions the transition from transient to steady-state is much more sudden (not shown). The complete cutoff energy fit in two dimensions is (where 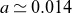 in the 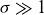 limit)
(4.3)

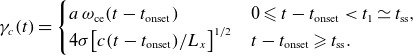




We also explicitly measure the high-energy cutoff at 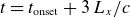 and find that it indeed adheres well to a linear scaling with 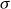 (panel *f*). We discuss the coefficient 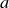 and comparisons with previous work in § 5.3.

### 4.3. Injection efficiencies

To contextualise the injection shares in the broader downstream population, we compute the injection (or number) efficiency, defined by
(4.4)

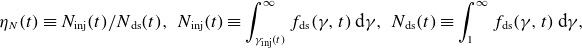

and the energy efficiency, defined by
(4.5)

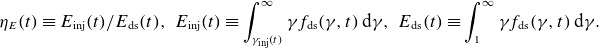

Since these quantities depend on 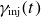 measured from time-evolving particle spectra, these efficiencies are well defined for 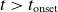 , i.e. when power-law spectra may be deciphered by our spectrum fitting procedure.

**Figure 6.**
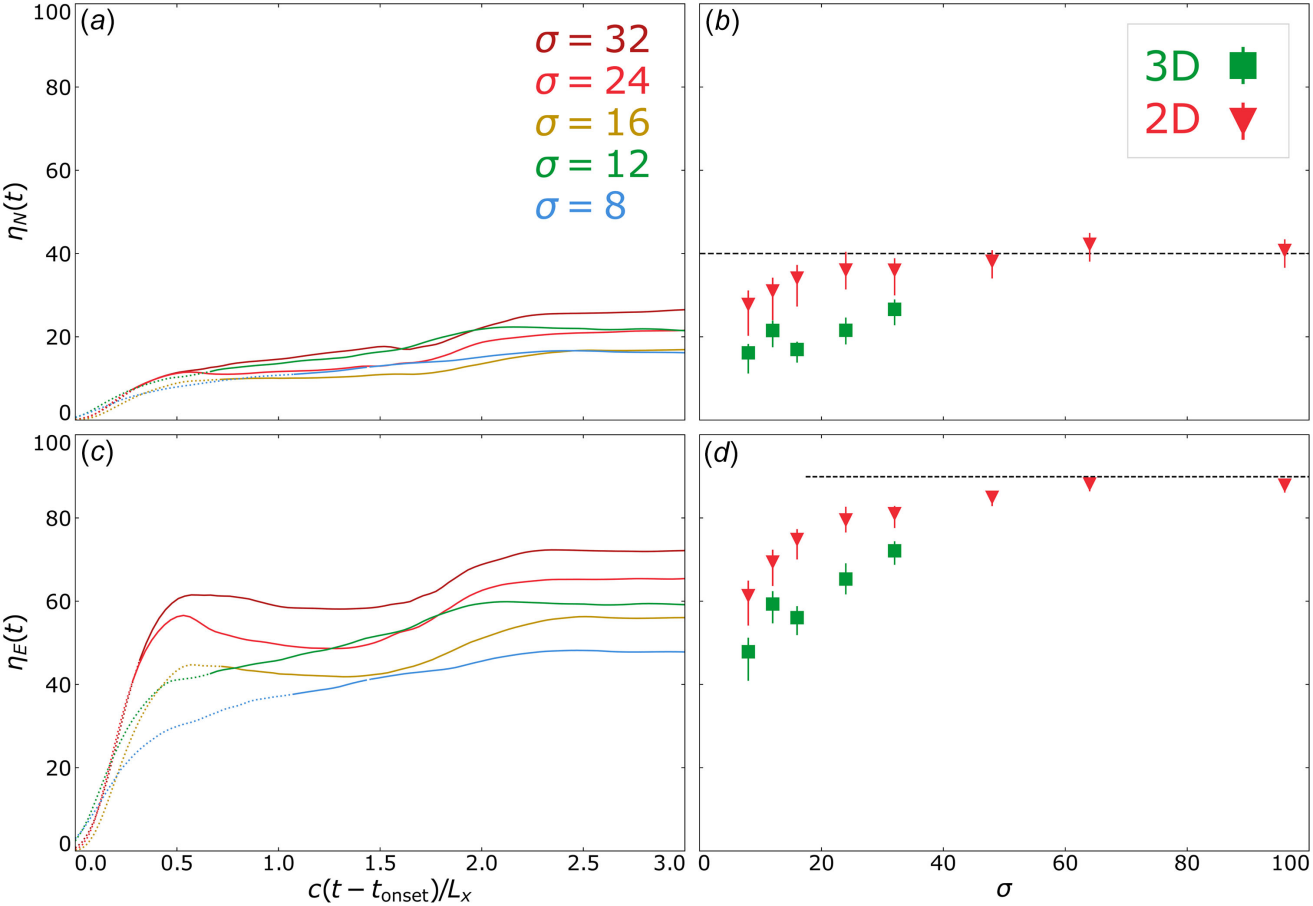
Efficiencies of particle injection and energy computed for 3-D simulations at various 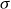 at each time step (*a*, *c*) and at final times (*b*, *d*), comparing 2-D (red triangle) and 3-D (green square) runs. Dotted segments in (*a*, *c*) indicate time steps for which the power-law extent was short (i.e. 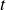 for which 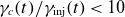 ), whereas solid lines indicate times where 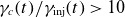 . (*a*, *b*) Injection efficiencies, where the dashed black horizontal line indicates 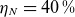 on (*b*). (*c*, *d*) Energy efficiencies, where the dashed black horizontal line indicates 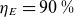 in (*d*). Time-evolved errors are not shown but are comparable to the final-time errors.

We use (4.4), (4.5) to directly compute 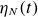 and 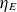 (t) for various 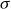 , as shown in figure 6.

We generally find that both 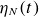 (panel a) and 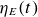 (panel c) display a period of rapid initial growth for approximately 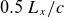 after 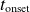 , slowing down significantly at intermediate times, eventually saturating at a finite 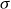 -dependent values 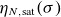 and 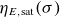 at late times, 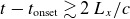 , in agreement with the slower late-time growth of 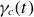 and stabilisation of 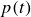 . In two dimensions, the time-saturated injection efficiency 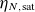 improves from 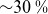 to 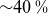 as 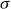 is increased from 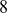 to 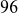 , at which point convergence with 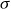 is achieved (panel b). The 3-D simulations systematically show somewhat lower values of 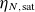 (20 %–25 %) than their 2-D counterparts for any given value of 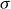 , likely owed to softer power-law indices; cf. figure 5. However, because of the limited 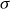 -range covered by our 3-D simulation campaign, it is difficult to extract any clear and reliable systematic trends for the dependence of 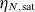 on 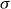 in three dimensions.

The final saturated energy efficiency 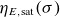 in 2-D simulations also grows monotonically starting from 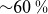 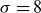 and saturates at the 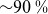 -level for 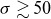 (panel d). In three dimensions, 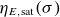 is again lower than in two dimensions, but, unlike 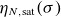 , exhibits clear monotonic growth with 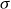 , exceeding 70 % at the highest probed value 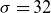 . Extending our 3-D study to even higher values of 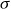 is clearly needed in order to determine how and what level 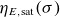 saturates in the ultra-relativistic limit 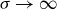 .

In summary, the time-converged injection and energy efficiencies become high ( 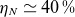 , 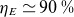 ) in two dimensions and are insensitive to 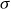 once it is sufficiently great. While these efficiencies are systematically lower in three dimensions likely due to softer power-law indices, they are not yet converged at 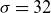 .

### 4.4. Injection shares

**Figure 7.**
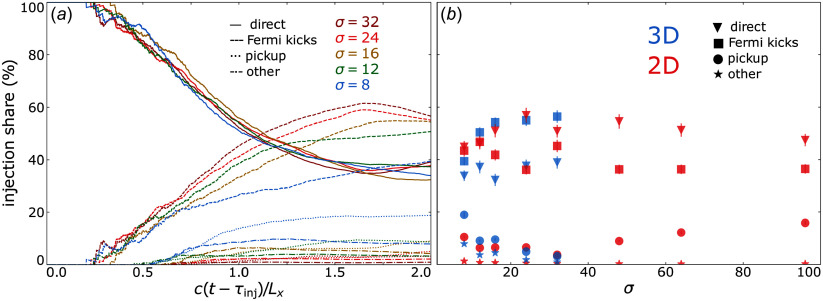
Contributions of each injection mechanism to the injected particle population. (*a*) Time-evolved injection shares from 3-D runs for several values of 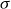 . (*b*) Injection shares over all runs at 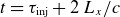 . All injection shares have an error of 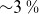 for each mechanism at every time step, propagated from errors of 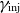 .

To obtain the time-evolved contribution of each injection mechanism (see § 2.2) to the total population of injected particles, we apply the formula
(4.6)

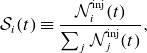

where 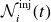 is the cumulative number of particles injected by mechanism 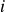 at time 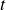 . We term these 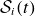 ’s ‘injection shares’ and plot them in figure 7 against 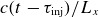 , where 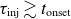 is the moment when the first injection of a tracer particle occurs.

In panel (*a*), we show time-evolved injection shares for 3-D simulations for the scanned values of 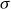 . Here, we find a specific activation sequence of the injection mechanisms that is consistent with our conceptual picture of injection in figure 1. Soon after 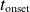 , the 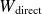 share is at 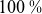 . At 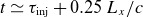 , Fermi kicks are activated and gradually (over 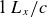 ) become the dominant injection mechanism (except for 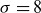 , where the pickup process takes a significant 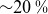 fraction). Finally, at 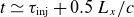 , particles start getting injected by the pickup mechanism, roughly coincident with the time when reconnection outflows are established. The time intervals between the activation of each subsequent mechanism appear independent of 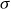 . While not shown in this figure, we also calculate the fraction of injected particles that are injected at these activation times: at 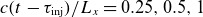 , only 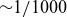 , 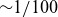 and 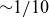 of particles that end up injected by 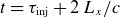 have been injected. This shows that the early dominance of direct acceleration may be misleading, since it represents 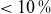 of the aggregate population of injected particles.

In panel (*b*), we plot the injection shares at 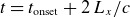 against 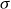 for 2-D and 3-D simulations. We find that injections by Fermi and direct acceleration remain competitive: Aside from 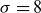 , we find in three dimensions that Fermi kicks contribute 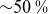 whereas direct acceleration contributes 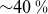 , and that these shares are flipped in two dimensions. Meanwhile, when increasing 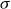 from 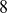 to 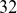 , we find that pickup shares are suppressed significantly, from 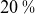 ( 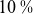 ) 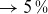 in three dimensions (two dimensions); however, they curiously rise back up in two dimensions when varying 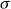 from 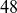 to 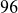 . A negligible ( 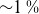 ) fraction of particles satisfy 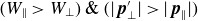 upon reaching the injection energy 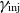 (categorised as ‘other’ in § 2.2), suggesting that any mechanisms which impart this combination onto particles can be ignored.

### 4.5. Non-thermal particle acceleration (NTPA) correlations of each mechanism

Given a particle of energy 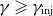 , what is the probability that it was injected by the mechanism 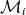 ? In other words, to what extent is each injection mechanism likely, by mere correlation, to produce particles of a given energy 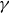 (e.g. 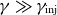 )? We attempt to answer this question by introducing the ‘NTPA correlation’ 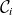 of each injection mechanism 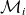 , defined by
(4.7)

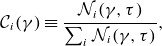

where 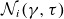 is the number of particles injected by mechanism 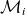 with energy 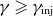 at a certain time 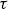 that will be taken to be 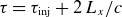 . We term these quantities ‘correlations’ to emphasise that the 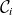 values merely represent the correlation between high-energy particles and their injection mechanism of origin.

**Figure 8.**
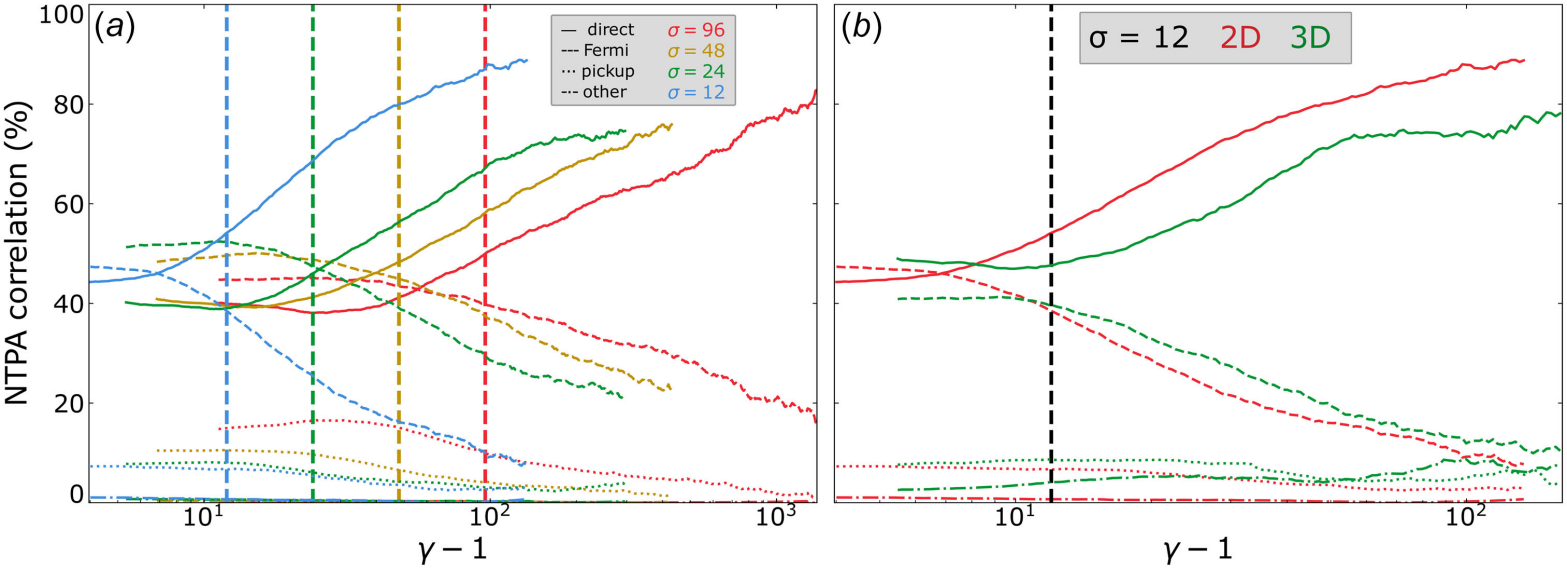
The NTPA correlation of each injection mechanism evaluated at 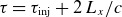 plotted against 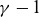 for 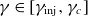 , with 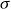 values indicated by the vertical dashed lines. (*a*) The NTPA correlations of four 2-D simulations with 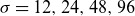 indicated by blue, green, gold and red lines, respectively. (*b*) The NTPA correlations of 2-D (red) and 3-D (green) 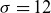 simulations.

To calculate 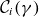 , we define 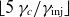 (i.e. rounded to the nearest integer below 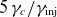 ) energy bins for 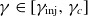 spaced uniformly in log space. Then, we populate these bins with the final energies particles attain, sorted by which mechanism injected them; this yields 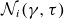 . Finally, we obtain 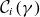 by normalising each histogram by 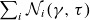 , thereby ensuring that 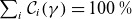 at each energy bin. In figure 8 we plot 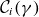 for each injection mechanism for 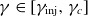 .

**Figure 9.**
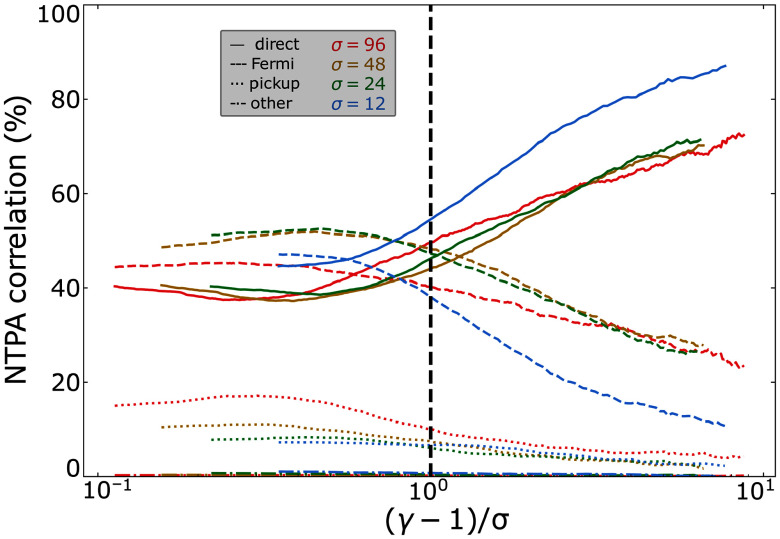
The NTPA correlation of each injection mechanism evaluated at 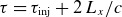 plotted against 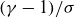 for 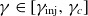 and 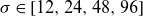 . The black dashed vertical line indicates 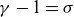 .

In figure 8(*a*), we compare the effects of magnetisation across four 2-D simulations with 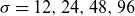 . We find that the more energetic a given particle is at the final time, the greater the likelihood that it was injected by 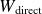 , with 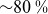 of particles around 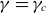 having been injected by this mechanism. Furthermore, it appears that 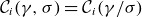 . To see this more clearly, we have plotted this separately in figure 9.

In figure 8(*b*), we compare two and three dimensions. Adding a third dimension appears to slightly dampen the direct acceleration correlations, with 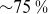 of particles at 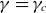 being injected by direct acceleration rather than 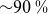 at 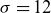 .

In summary, despite direct acceleration injecting only 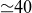 %– 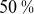 of particles, there is a 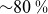 NTPA correlation between the particles with 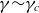 and injection by direct acceleration. These results may explain some of the disparities in the literature on particle injection in the weak guide field ( 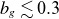 ) regime, wherein Totorica *et al*. (2023) and Gupta *et al*. (2025) found direct acceleration to dominate NTPA correlations and French *et al*. (2023) found Fermi kicks to dominate injection shares. We elaborate further in § 5.2.

## 5. Discussion

### 5.1. The injection criterion

Historically, the meaning of the term injection in the published literature on relativistic magnetic reconnection has varied. The works by Ball *et al*. (2019), Kilian *et al*. (2020) and French *et al*. (2023) consider injection to be the energisation of particles up until the low-energy bound of the power-law spectrum. Meanwhile, Sironi (2022), Totorica *et al*. (2023) and Gupta *et al*. (2025) describe injection as the ‘early stages’ of particle acceleration. Nevertheless, each concept has been made rigorous in a similar fashion by:

- defining ‘injection’ as the acceleration that occurs up to a threshold energy 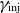 (Ball *et al*. 2019; Kilian *et al*. 2020; Sironi 2022; French *et al*. 2023; Totorica *et al*. 2023; Gupta *et al*. 2025); and
- modelling this threshold as 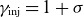 (Ball *et al*. 2019; Kilian *et al*. 2020; Sironi 2022) or 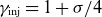 (Sironi 2022; Totorica *et al*. 2023; Gupta *et al*. 2025).

While we (in the present work and in French *et al*. (2023)) retained convention (i), i.e. that there exists a certain characteristic energy 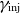 , surpassing which can serve as the criterion for injection, we discarded assumption (ii), opting instead to measure 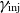 from particle spectra directly. Our procedure for measuring 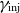 (cf. Appendix B and French *et al*. (2023)) is guided by the first conception mentioned above, i.e. injection as the energisation of particles up until the low-energy bound of the non-thermal power-law spectrum.

Our measurements in the present work (where 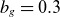 and 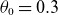 ) have revealed that the 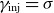 model vastly overestimates the low-energy bound of the power-law spectrum in both two and three dimensions (figure 5 *d*). In two dimensions, the 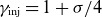 model still overestimates measurements for 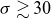 ; however, in three dimensions we lack the data at such high values of 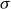 to make the same conclusion.

The 2-D measurements are, however, consistent with our model for the injection criterion, which defines a particle as injected if its gyroradius 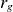 exceeds the elementary CS half-thickness 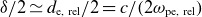 (cf. § 2.1). In the thermally dominated regime, this yields an injection energy 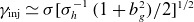 . In particular, this model is consistent with our measurements of the upstream magnetisation dependence of 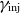 in two dimensions (figure 5 *d*). In three dimensions, however, the model systematically underestimates the measurements, which we speculate is owed to CSs disrupting at greater thicknesses in three dimensions.

A potentially important implication of this model is that if no particles can achieve 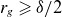 , then no particles are injected and thus a power-law spectrum of non-thermal particles does not form. Therefore, if one can determine *a priori* whether any particles can be injected, one could determine precisely, from system parameters alone, the conditions under which a power-law spectrum can form. A future study could test this systematically by (a) varying 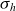 from 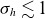 to 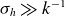 , (b) estimating the injection energy and the work done by each mechanism over this parameter range and (c) seeing whether the formation of a power-law spectrum coincides with the ability for at least one mechanism to inject particles. Moreover, a future study could examine the effects of using an electron–ion plasma, where the two-tiered structure of CSs may have a significant effect on the injection energy and relevant injection mechanisms for each species.

While the present study focuses on a single guide-field strength 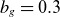 , the injection criterion developed in § 2.1 provides insight into the expected guide-field dependence of 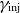 . The explicit factor of 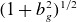 in (2.4) implies that stronger guide fields increase the injection energy, as the enhanced total magnetic field strength reduces the particle gyroradius and thus requires greater particle energy to satisfy the criterion 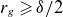 . For weak guide fields ( 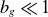 ), 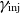 is insensitive to 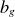 , whereas for strong guide fields ( 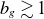 ), 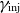 grows approximately linearly with 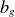 . This scaling is roughly consistent with measurements in French *et al*. (2023), which focused on 2-D reconnection and found that 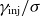 increases from 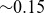 at 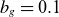 to 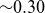 at 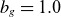 . A systematic study that compares measurements of 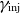 over varying both 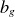 and 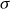 in 3-D relativistic reconnection remains a direction for future work.

### 5.2. The injection shares and NTPA correlations

In addition to the injection criterion having differing conceptions in the literature (§ 5.1), there have also been differing conceptions about how to evaluate the relative importance of various injection mechanisms. In this work and French *et al*. (2023), we consider injection shares – i.e. the fractional contribution of each mechanism to the total injected particle population – to reflect that mechanism’s importance. In contrast, works by Totorica *et al*. (2023) and Gupta *et al*. (2025) essentially^3^ consider NTPA correlations, evaluated at the highest permissible energies, to reflect mechanism importance.

Despite these studies evaluating different mechanisms, we may nevertheless make a comparison with the NTPA correlations evaluated in this study. Indeed, we also find that the higher the final particle energy, the greater the likelihood (approaching 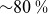 ) that the particle was injected by direct acceleration, which is often associated with non-ideal (including 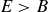 ) electric fields when 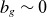 (figures 8, 9). However, while NTPA correlations at high energies [e.g. 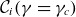 ] can certainly be quite useful in their own right, they have little bearing on the dominant injection mechanism. This is because particles with a head start in entering the power law – such as those injected by direct acceleration, which dominates at early times as suggested by figure 7 – will participate in the continual Fermi mechanism that operates in the power law for a longer duration, allowing them to achieve the highest energies. This significantly biases 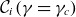 as a metric for evaluating particle injection, since the particles injected at times closer to 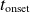 are over-represented.

Conversely, the injection shares can be read off figure 2(*b*) of Totorica *et al*. (2023) by evaluating each line at 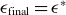 , which yields 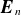 contributing 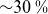 to the injected particle population for the threshold 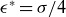 for reconnection with 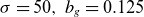 ; this is somewhat consistent with our previous work which found 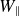 to contribute 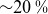 for the threshold 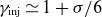 when 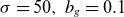 (French *et al*. 2023). Likewise, the injection shares can be read off figure 3 of Gupta *et al*. (2025) by evaluating each line at 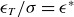 , which shows that 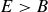 electric fields inject 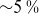 of particles, in rough agreement with Guo *et al*. (2023).

The guide-field strength is also expected to affect the relative contributions of the injection mechanisms. As shown in (2.13) and (2.14), both Fermi kicks and pickup acceleration are suppressed by factors containing 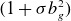 in the denominator. Consequently, for weak guide fields ( 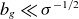 ), these mechanisms associated with the motional (perpendicular) electric field can efficiently inject particles, whereas for strong guide fields ( 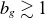 ), their energisation is significantly damped. In the strong guide-field regime, direct acceleration by the parallel electric field – which is not suppressed by the guide field – is expected to dominate the injection process. This trend is consistent with French *et al*. (2023), which found that 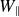 contributions increase from 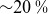 to 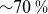 as 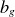 increases from 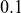 to 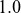 .

### The high-energy cutoff 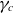


5.3.

Power-law spectra are often modelled with an exponential cutoff, i.e. 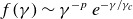 , where 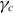 is the high-energy cutoff. We will discuss here two questions about this quantity: (a) How does 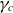 evolve with time? (b) How does 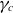 vary with upstream magnetisation 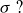


Let us begin with the first question. The time evolution of 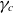 can be roughly decomposed into three phases: (i) the short, transient phase of rapid rise which immediately follows reconnection onset ( 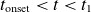 ), (ii) an intermediate phase, characterised by a mixed reconnection layer that is populated with both CSs and developing plasmoids ( 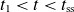 ), and (iii) the slow, quasi-steady phase, characterised by a fully developed multi-plasmoid dynamics in the reconnection layer ( 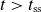 ). We show how 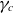 evolves during these phases in three dimensions for several values of 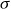 in figure 5(*e*) (§ 4.2).

To our knowledge, no dedicated study has yet numerically explored the evolution 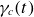 systematically during phase (i). Our high-cadence depositions of particle spectra have enabled us to explore this for the first time in detail. We find that this phase lasts approximately 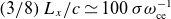 ; our measurements of 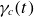 adhere closely to the simple linear form 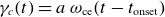 in both two and three dimensions, where 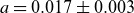 is a fitting parameter. This scaling of 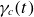 agrees with the time-dependent work done by the reconnection electric field, 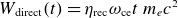 , except somewhat smaller in magnitude ( 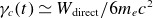 ). This agreement conveys a narrative consistent with the injection shares, which suggest that direct acceleration in small, elementary diffusion regions around X-points is the exclusive injector of particles during the first 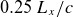 following reconnection onset (figure 7). The discrepancy of 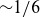 may be attributed to the fact that particles with finite drift in the 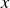 – 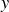 plane will leave elementary diffusion regions around X-points on microscopic time scales.

The existence of an intermediate phase in three dimensions where 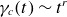 for 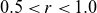 may be attributed to particles that have escaped plasmoids (e.g. disrupted by the flux-rope kink instability) and enter Speiser-like orbits which energise them as 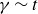 (Uzdensky *et al*. 2011; Cerutti *et al*. 2012, 2014; Dahlin, Drake & Swisdak 2017; Zhang *et al*. 2021). As plasmoids develop in the reconnection layer, the relative population of ‘trapped’ ( 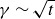 ) to ‘free’ ( 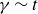 ) particles increases, eventually leading to the steady-state phase wherein 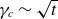 .

During the subsequent quasi-steady phase (i.e. 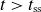 ), previous 2-D studies by Petropoulou & Sironi (2018) and Hakobyan *et al*. (2021) discovered that the high-energy cutoff grows as 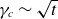 due to the adiabatic compression of plasmoids. This scaling of 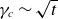 has also been found in three dimensions in the transrelativistic 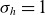 regime by Werner & Uzdensky (2021). Our results reaffirm this scaling in both two and three dimensions, where the measured 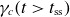 adheres closely to the model 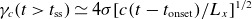 in two dimensions (not shown) and 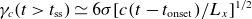 in three dimensions (figure 5 *e*). Our work in three dimensions does not, however, access the high- 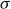 , large- 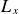 regime wherein secondary power laws of 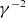 at higher energies have been found (Zhang *et al*. 2023).

The second question of how 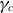 varies with 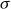 was originally explored by Werner *et al*. (2016), who found 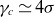 in two dimensions. We also find 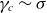 in both two and three dimensions but with a time-dependent coefficient in accordance with Petropoulou & Sironi (2018) (figure 5 *f*).

Combining our understanding of these two questions allows us to construct a more complete description of the high-energy cutoff evolution in 3-D relativistic magnetic reconnection (4.2).

## 6. Conclusions

The primary goal of this work was to understand energetic particle injection in relativistic magnetic reconnection – a poorly understood yet essential aspect of reconnection-driven non-thermal particle acceleration. Therefore, we sought to elucidate (i) what is and what sets the injection criterion and (ii) what physical mechanisms promote the particles to satisfy the criterion.

More concretely, we performed an array of PIC simulations of a reconnecting relativistic collisionless pair plasma immersed in a weak guide field of 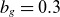 . From these simulations, we (i) measured the injection energy 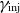 (§ 4.2) and (ii) evaluated the time-dependent contributions of three distinct acceleration mechanisms to the total injected particle population (§ 4.4), and investigated how these quantities depend on the upstream magnetisation 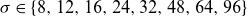 and dimensionality (two vs three dimensions). We have also devised a physical model for 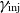 (§ 2.1) which is consistent with 2-D results.

This work also had the secondary goals of (i) evaluating the injection and energy efficiencies 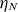 and 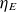 (§ 4.3) and (ii) uncovering the NTPA correlations of each mechanism, i.e. the correlations between injected particle energy and injection mechanism of origin (§ 4.5). We summarise our findings in the rest of this section.

### 6.1. Characteristic spectral parameters and efficiencies

Using a novel procedure for fitting power-law spectra, we have been able to acquire the three key spectral parameters – power-law index 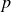 , injection energy 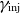 , and cutoff energy 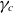 (from 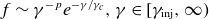 ) – with unprecedented precision (see Appendix B for details). This has enabled us to assert the following conclusions:

- Injection energy 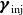 : the injection energy 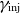 (i.e. the energy at which the power-law component of the downstream particle spectrum begins) grows sub-linearly with increasing magnetisation 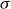 . In particular, 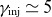 to 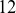 when varying 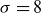 to 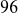 in two dimensions and 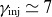 to 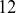 when varying 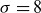 to 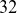 in three dimensions; 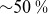 greater than 2-D measurements for each value of 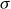 .
  The 2-D measurements of 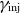 adhere well to the model we developed for the injection criterion, specifically in the thermally dominated regime (i.e. the intermediate 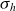 regime whereupon the plasma temperature inside elementary CSs is governed by the ambient upstream plasma), wherein the injection energy is 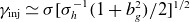 (§ 2.1, figure 5 *d*). In contrast, in three dimensions the model consistently underestimates the measurements by a factor of 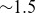 .
- Power-law index 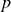 : after reconnection onset, power-law spectra rapidly harden to 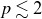 and then gradually soften, stabilising at 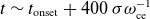 (figure 5 *a*). At final times (i.e. 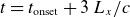 ), the power-law index 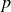 declines with increasing 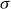 , varying from 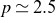 to 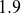 when varying 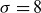 to 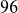 in two dimensions and 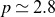 to 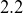 when varying 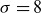 to 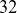 in three dimensions. Power-law spectra are consistently steeper by 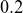 – 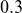 in three dimensions compared with two dimensions for the same value of 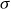 (figure 5 *b*).
- Cutoff energy 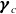 : during the first 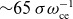 following reconnection onset, virtually the only injection mechanism that is active is direct acceleration by 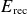 in non-ideal reconnection diffusion regions (cf. figure 7 *a*). Hence we sought to test whether during its initial rise 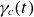 would evolve similarly to 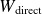 , i.e. 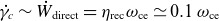 . Indeed, we found that 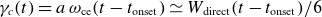 closely matches the 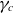 measurements during the early transient period 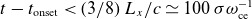 (figure 5 *e*). After this transient period, we find that 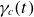 transitions to a slower-growth second stage, adhering closely to 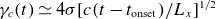 in two dimensions and 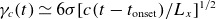 in three dimensions, in agreement with previous work (cf. § 5.3), see figure 5(*e*).
- Injection efficiency 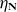 and energy efficiency 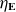 : the ‘efficiencies’ of injection 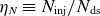 ((4.4); i.e. the fraction of the downstream particles that are injected) and energy 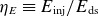 [(4.5); i.e. the fraction of the downstream particle kinetic energy carried by injected particles] grows rapidly during the first 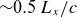 that follows reconnection onset (figure 6 *a*, *c*). Subsequently, these efficiencies continue to grow throughout the simulation, but slower, and after 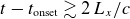 saturate at finite, 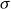 -dependent values 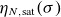 and 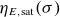 .
  The late-time saturated injection efficiency 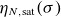 increases with 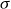 in both two and three dimensions. In particular, in two dimensions it grows from approximately 30 % to 40 % as 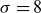 to 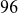 , while in three dimensions it increases from approximately 15 % to 25 % as 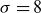 to 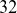 (figure 6 *b*, *d*). Likewise, the time-saturated energy efficiency 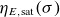 also grows with 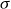 , increasing from 60 % to 90 % as 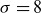 to 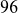 in two dimensions and from 50 % to 70 % as 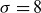 to 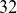 in three dimensions.
  In two dimensions, the 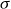 -convergence of the efficiencies around 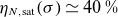 and 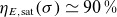 is realised for 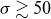 , whereas in three dimensions such convergence is not yet established, likely due to the limited 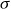 -range covered by our 3-D simulations. These 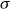 -converged 2-D measurements are in agreement with our previous study (French *et al*. 2023). The consistently lower values of 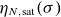 , 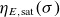 in three dimensions are likely owed to the steeper power-law spectra found in three dimensions (figure 5 *b*).

### 6.2. Injection mechanism shares and NTPA correlations

- Injection shares: we find a specific activation sequence of the injection mechanisms that agrees with our geometrical picture of injection (figure 1). Denoting the time of the first tracer particle injection as 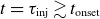 , the first mechanism to activate is 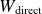 at 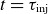 , followed by 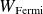 at 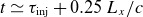 , followed by 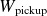 at 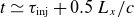 (figure 7 *a*). Importantly, of all the particles that are injected by the end of each simulation, only 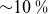 are injected during the initial 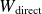 -dominated phase (i.e. 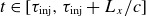 ). This implies that while 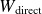 is initially important, it can nevertheless become sub-dominant when evaluating the aggregate injected particle population over a longer period.
  The asymptotic, late-time injection shares from Fermi kicks and direct acceleration trend toward 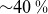 and 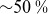 respectively in three dimensions (two dimensions) with increasing 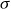 (figure 7 *b*). Pickup shares are suppressed from 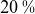 ( 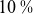 ) to 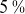 in three dimensions (two dimensions) when varying 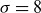 to 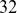 , but rise again in two dimensions when varying 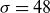 to 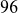 .
- NTPA correlations: to discover the probability that a given particle of high energy 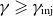 was injected by a certain mechanism, we compute ‘NTPA correlations’ (figure 8). We find that particles injected by 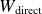 comprise a majority of particles with 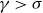 and comprise 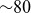 – 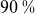 of particles with 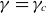 . Introducing a third dimension alters this result, with the correlation between direct acceleration and particles at 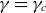 falling from 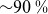 to 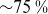 at 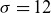 .

This work advances our understanding of particle injection in the relativistic regime of magnetic reconnection in several ways. By systematically comparing 2-D and 3-D simulations across a broad range of upstream magnetisations, we found that 3-D effects increase injection energies by 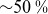 and that the injection energy 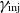 scales sub-linearly with upstream magnetisation 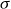 when the hot upstream magnetisation 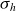 is moderate. The theoretical picture of particle injection presented in this work provides a physical basis for understanding how particles enter continual acceleration within the power-law spectrum produced by magnetic reconnection and is consistent with measurements of injection energies in two dimensions. Moreover, by distinguishing between injection shares (quantifying the contribution of each mechanism to the injected particle population) and NTPA correlations (which mechanisms injected the particles that reach the highest energies), this work reconciles apparent contradictions in the literature on particle injection in relativistic magnetic reconnection. Finally, the time-resolved analysis of injection mechanism activation sequences and the precise spectral fitting procedure developed here establish a framework for future studies of particle injection across different plasma conditions. These results have direct implications for modelling high-energy emissions from astrophysical reconnection sites, where the existence of a thermally dominated regime implies that power-law spectra produced by magnetic reconnection in the high 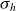 limit can potentially be much broader than previously expected.

## Figures and Tables

**Figure 10.**
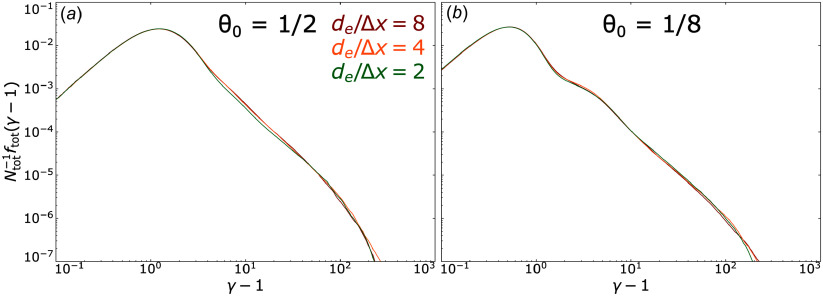
Particle spectra for different resolutions. (*a*) Uses an ambient upstream temperature of 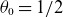 whereas (*b*) uses 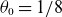 .

**Figure 11.**
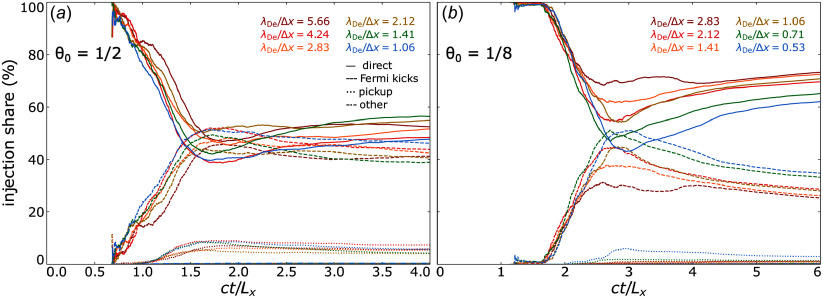
Injection shares for different resolutions. In each panel, the corresponding resolution in skin depths is 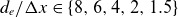 from dark red to blue lines. (*a*) Uses an ambient upstream temperature of 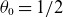 whereas (*b*) uses 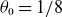 .

**Figure 12.**
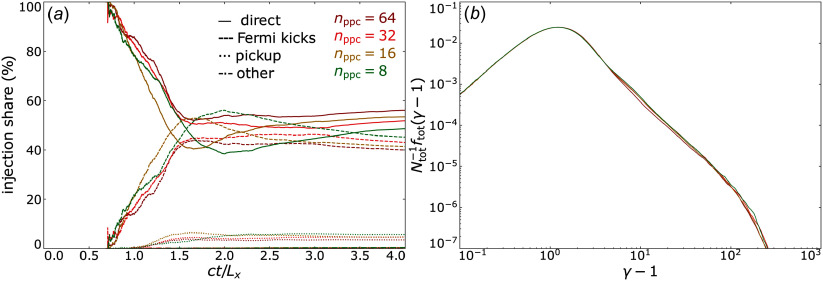
(*a*) Particle spectra for different 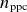 . (*b*) Injection shares for different 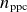 .

**Figure 13.**
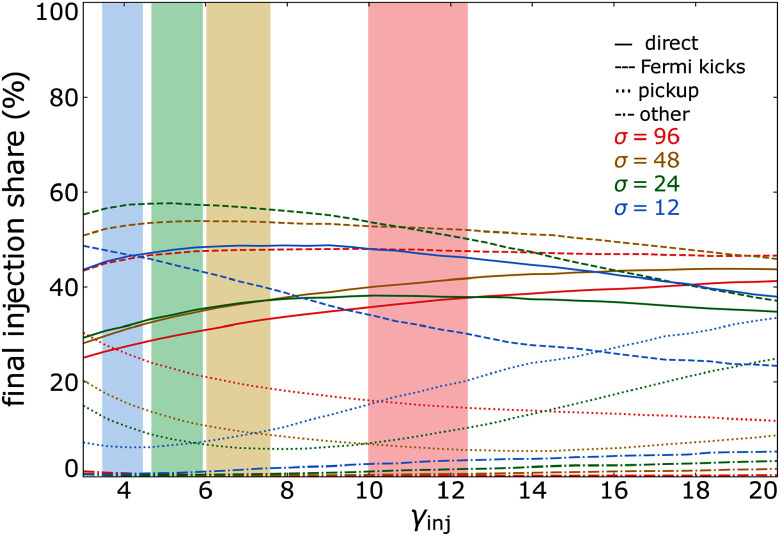
Injection shares at 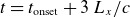 as a function of 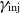 for 2-D simulations. The solid bars indicate the error about the measured injection energies.

## Footnotes

1
Previous work has found that introducing a perturbation with  significantly influences 3-D results; in particular, making them more similar to 2-D results which are insensitive to a perturbation (Werner & Uzdensky 2021). However, when performing a similar test using a guide magnetic field of strength  , we find that the 3-D results (energy evolution, injection shares, particle spectra and reconnection rates) are insensitive to a perturbation, possibly because of the stabilising effect of the guide field that already makes 3-D results more similar to 2-D results.

2
By contrast, in three dimensions particles may escape the downstream and, from the perspective of the mixing diagnostic, proceed to ‘contaminate’ the upstream, leading to the small portions of the upstream being mis-identified as downstream. To combat this issue a lower mixing threshold may be used, which in turn, however, may mis-identify part of the downstream as upstream. Considering these two issues, a recent study has found the optimal mixing ratio threshold in three dimensions to be 70 % (Zhang *et al*. 2021).

3
The assessment of mechanism contributions done in Totorica *et al.* (2023) and Gupta *et al.* (2025) differs from the NTPA correlations defined in § 4.5 only in that, instead of quantifying the contributions by the discrete metric of the number of particles they each inject, in those works they are quantified by the overall amount of work they do on particles with  . This is possible because these studies consider mechanisms characterised by time-integrated quantities (i.e. work done by ideal vs non-ideal electric fields in Totorica *et al.* (2023) and work done by  vs  electric fields in Gupta *et al.* (2025)). For a statistically large sample of tracer particles, the shares defined by the number of particles injected by a mechanism should approach those defined in terms of the overall work done on particles with energies  by that mechanism.

## Appendix A. Convergence studies

In general when conducting PIC simulations, it is crucial to have robust results, and as such one aims to use a domain size as large as possible. However, since one is constrained by the computational resources that exist, it is important to consider the quantities which control the computational time. For a given simulation code, the computational cost  scales with the number of particle pushes, given by  , where  is the number of cells in the domain,  is the number of pushes, and  is the number of particles per cell.

Since we are preserving aspect ratios to  (  ) in three dimensions (two dimensions), the cost reduces to  in two dimensions and  in three dimensions. In particular, for a given domain size  we may cast  in terms of spatial resolution  , where  is a local length scale and  is the grid length. These scalings motivate using values of  and  that are as low as possible while producing results (e.g. particle spectra, injection shares, injection efficiencies, etc.) which are (a) approximately invariant to increases of these inputs and (b) sensitive to reductions of these inputs. The parameters we have decided to use for these convergence studies are summarised in table 1.

**Table 1.** Quantities for convergence studies.

		Particles per cell
Quantity	Resolution convergence study	convergence study
Domain size ( )		
Upstream magnetisation ( )		
# of cells in ( )		
# ptl per cell per species ( )		
Resolution ( )		
Guide-field strength ( )		
Mixing fraction ( )		
# of tracer particles ( )		
Ambient upstream temp. ( )		
Thickness of initial CS ( )	for	
Running time ( )	for	
Drift velocity ( )	for	
Aspect ratio ( )		
Number of cells in ( )		

To save computational resources, we will conduct these convergence studies in two dimensions and extend the outcomes to three dimensions as follows. For resolution convergence, we will assume that the minimum spatial resolution necessary to resolve in three dimensions is identical to two dimensions. For  convergence, we will assume that the number of particles per Debye  -sphere remain fixed, so that  , allowing us to straightforwardly solve for  .

### A.1 Resolution convergence study

The particular length scale  which must be resolved for converged results is not obvious, and therefore we shall experiment with different scales. In order to satisfy the CFL (Courant, Friedrichs & Lewy 1928) condition, it is necessary to forbid particles moving at light speed from traversing more than one grid length  per time step  , i.e.  , where  is the collisionless relativistic electron skin depth. Therefore the first length scale we will test is  , i.e. we seek to determine whether there exists some critical resolution  above which results vary negligibly and below which results are sensitive.

Additionally, we will conduct a parallel set of simulations that use a different initial upstream background temperature  . This is done to uncover which value of  which must be resolved. This is also done to determine whether there exists a regime of  for which all particle injection results are approximately invariant. This is of interest not only to reveal increased generality of results, but also potentially to save computational resources, because (a) using  yields larger values  , (b) it means that a larger drift speed  can be implemented in the initial current layer without initiating the two-stream instability, thereby causing reconnection to start earlier, and (c) the initial CS is thinner, causing it to occupy a smaller fraction of the total particle population for a given length in the  -direction  , thereby yielding more precise results for tracer particle analysis.

In this (2-D) convergence study we preserve the number of particles per Debye volume, so that doubling the resolution folds  by a factor of 4.

Regardless of whether the background temperature is  or  , we find that the particle spectra are invariant of resolution, implying that all resultant spectral quantities (  ) are converged (figure 10). Furthermore, while the injection energy (i.e. the low-energy bound of the power-law spectrum) may be difficult to visually distinguish from the thermal component when  (panel *a*), it becomes clear when  that  .

We show the influence of spatial resolution on the particle injection shares in figure 11. In order to display only statistically significant results, we only show injection shares after  of the total injected tracer particle population has undergone injection. We find that the injection shares have more difficulty converging, with no clear monotonic trend emerging upon varying  . However, we find that  causes a significant break in the results (panel b).

### A.2 Particles per cell convergence study

In this study we strive to find a definite  (number of particles per cell per species) above which the results are insensitive and below which the results are sensitive. We find negligible variation for the injection energies and efficiencies, owing to well-converged spectra (figure 12 *b*). However, when checking injection shares, we find a significant difference between  and  , especially for  (figure 12 *a*). Meanwhile,  yield similar injection shares for all mechanisms, remaining within  during the entire evolution. Therefore, we determine that  captures the results of greater  . Preserving the number of particles per Debye volume, this implies that we use  in 3-D simulations.

## Appendix B. Spectral fitting procedure

To measure the injection energies, we identically follow the fitting procedure described in French *et al.* (2023) bar the following improvements. The first is that, rather than scanning over a range of  values, we set  to twice the energy of the spectral peak of the downstream particles. This simplifies the procedure and reduces the number of inputs without incurring much difference to the results. Second, instead of setting ‘the’ power-law index as the median of the collection of power-law indices that fall within a certain tolerance, we collect the median power-law index over the interval (denoted  ) and plug in power-law indices  around it  into the expression

(B1)

where  and  are the energies that bound the longest logarithmic power-law segment that resides within the power-law tolerance. The goal is to determine by brute force the  that yields the minimum  in the set  . Notice that  is defined to ensure that the fit intersects  exactly once. We choose  because that is sufficiently large to capture the best power-law index available.

Owing to these improvements to the fitting procedure, we are able to employ stricter power-law tolerances,  and a smaller  (where  is the local power-law index) to define the injection energy. By contrast, in our previous work we used  and  (French *et al.* 2023). The measurement errors of the power-law indices are within  , injection energies  within  , and high-energy cutoffs  within  .

To show the insensitivity of the choice of  on the final injection shares, we show the final injection shares against  in figure 13. From the negligible variation of the final injection shares over each shaded bar, we see that regardless of whether  is chosen to be  (as in this work) or  as in French *et al.* (2023), the injection shares are not significantly altered.

## References

[ref1] Ball, D. , Sironi, L. & Özel, F. 2019 The mechanism of electron injection and acceleration in transrelativistic reconnection. ApJ 884, 57.

[ref2] Blackman, E.G. & Field, G.B. 1994 Kinematics of relativistic magnetic reconnection. Phys. Rev. Lett. 72, 494–497. 10056447 10.1103/PhysRevLett.72.494

[ref3] Blandford, R. & Eichler, D. 1987 Particle acceleration at astrophysical shocks: a theory of cosmic ray origin. Phys. Rep. 154, 1–75.

[ref4] Blandford, R.D. & Ostriker, J.P. 1978 Particle acceleration by astrophysical shocks. ApJL 221, L29–L32.

[ref5] Bulanov, S.V. & Sasorov, P.V. 1976 Energy spectrum of particles accelerated in the neighborhood of a line of zero magnetic field. Soviet Ast. 19, 464–468.

[ref6] Caprioli, D. & Spitkovsky, A. 2014 Simulations of ion acceleration at non-relativistic shocks. i. acceleration efficiency. Astrophys. J. 783, 91.

[ref7] Cerutti, B. , Uzdensky, D.A. & Begelman, M.C. 2012 Extreme particle acceleration in magnetic reconnection layers: application to the gamma-ray flares in the crab nebula. Astrophys. J. 746, 148.

[ref8] Cerutti, B. , Werner, G.R. , Uzdensky, D.A. & Begelman, M.C. 2013 Simulations of particle acceleration beyond the classical synchrotron burnoff limit in magnetic reconnection: an explanation of the crab flares. ApJ 770, 147.

[ref9] Cerutti, B. , Werner, G.R. , Uzdensky, D.A. & Begelman, M.C. 2014 Three-dimensional relativistic pair plasma reconnection with radiative feedback in the crab nebula. Astrophys. J. 782, 104.

[ref10] Chandran, B.D.G. 2000 Scattering of energetic particles by anisotropic magnetohydrodynamic turbulence with a goldreich-sridhar power spectrum. Phys. Rev. Lett. 85, 4656–4659. 11082620 10.1103/PhysRevLett.85.4656

[ref11] Chernoglazov, A. , Hakobyan, H. & Philippov, A. 2023 High-energy radiation and ion acceleration in three-dimensional relativistic magnetic reconnection with strong synchrotron cooling. Astrophys. J. 959, 122.

[ref12] Comisso, L. & Sironi, L. 2018 Particle acceleration in relativistic plasma turbulence. Phys. Rev. Lett. 121, 255101. 30608827 10.1103/PhysRevLett.121.255101

[ref13] Comisso, L. & Sironi, L. 2019 The interplay of magnetically dominated turbulence and magnetic reconnection in producing nonthermal particles. Astrophys. J. 886, 122.

[ref14] Courant, R. , Friedrichs, K. & Lewy, H. 1928 Über die partiellen differenzengleichungen der mathematischen physik. Math. Ann. 100, 32–74.

[ref15] Dahlin, J.T. , Drake, J.F. & Swisdak, M. 2014 The mechanisms of electron heating and acceleration during magnetic reconnection. Phys. Plasmas 21, 092304.

[ref16] Dahlin, J.T. , Drake, J.F. & Swisdak, M. 2017 The role of three-dimensional transport in driving enhanced electron acceleration during magnetic reconnection. Phys. Plasmas 24, 092110.

[ref17] Daughton, W. , Nakamura, T.K.M. , Karimabadi, H. , Roytershteyn, V. & Loring, B. 2014 Computing the reconnection rate in turbulent kinetic layers by using electron mixing to identify topology. Phys. Plasmas 21, 052307.

[ref18] Demidem, C. , Lemoine, M. & Casse, F. 2020 Particle acceleration in relativistic turbulence: a theoretical appraisal. Phys. Rev. *D* 102, 023003.

[ref19] Drake, J.F. , Cassak, P.A. , Shay, M.A. , Swisdak, M. & Quataert, E. 2009 A magnetic reconnection mechanism for ion acceleration and abundance enhancements in impulsive flares. Astrophys. J. 700, L16–L20.

[ref20] Drake, J.F. , Swisdak, M. , Che, H. & Shay, M.A. 2006 Electron acceleration from contracting magnetic islands during reconnection. Nature 443, 553. 17024088 10.1038/nature05116

[ref21] Egedal, A.L. & Daughton, W. 2013 A review of pressure anisotropy caused by electron trapping in collisionless plasma, and its implications for magnetic reconnection. Phys. Plasmas 20, 061201.

[ref22] Fermi, E.N.R.I.C.O. 1949 On the origin of the cosmic radiation. Phys. Rev. 75, 1169–1174.

[ref23] French, O. , Guo, F. , Zhang, Q. & Uzdensky, D.A. 2023 Particle injection and nonthermal particle acceleration in relativistic magnetic reconnection. Astrophys. J. 948, 19.

[ref24] Giannios, D. , Uzdensky, D.A. & Begelman, M.C. 2010 Fast teV variability from misaligned minijets in the jet of m87. Mon. Not. R. Astron. Soc. 402, 1649–1656.

[ref25] Goodbred, M. & Liu, Y.-H. 2022 First-principles theory of the relativistic magnetic reconnection rate in astrophysical pair plasmas. Phys. Rev. Lett. 129, 265101. 36608210 10.1103/PhysRevLett.129.265101

[ref26] Guo, F. , French, O. , Zhang, Q. , Li, X. & J., Seo . 2025 Particle injection problem in magnetic reconnection and turbulence. arXiv: 2506.19938.

[ref27] Guo, F. , Li, H. , Daughton, W. & Liu, Y.-H. 2014 Formation of hard power laws in the energetic particle spectra resulting from relativistic magnetic reconnection. Phys. Rev. Lett. 113, 155005. 25375716 10.1103/PhysRevLett.113.155005

[ref28] Guo, F. , Li, X. , Daughton, W. , Kilian, P. , Li, H. , Liu, Y.-H. , Yan, W. & Ma, D. 2019 Determining the dominant acceleration mechanism during relativistic magnetic reconnection in large-scale systems. ApJ 879, 5.

[ref29] Guo, F. , Li, X. , French, O. , Zhang, Q. , Daughton, W. , Liu, Y.-H. , Matthaeus, W. , Kilian, P. , Johnson, G. & Li, H. 2023 Comment on ‘nonideal fields solve the injection problem in relativistic reconnection. Phys. Rev. Lett. 130, 189501. 37204887 10.1103/PhysRevLett.130.189501

[ref30] Guo, F. , Li, X. , Li, H. , Daughton, W. , Zhang, B. , Lloyd-Ronning, N. , Liu, Y.-H. , Zhang, H. & Deng, W. 2016 Efficient production of high-energy nonthermal particles during magnetic reconnection in a magnetically dominated ion–electron plasma. Astrophys. J. 818, 7.

[ref31] Guo, F. , Liu, Y.-H. , Daughton, W. & Li, H. 2015 Particle acceleration and plasma dynamics during magnetic reconnection in the magnetically dominated regime. ApJ 806, 167.

[ref32] Guo, F. , Liu, Y.-H. , Zenitani, S. & Hoshino, M. 2024 Magnetic reconnection and associated particle acceleration in high-energy astrophysics. Space Sci. Rev. 220, 43.

[ref33] Gupta, S. , Sridhar, N. & Sironi, L. 2025 The role of electric dominance for particle injection in relativistic reconnection. MNRAS 538, 49–59.

[ref34] Hakobyan, H. , Petropoulou, M. , Spitkovsky, A. & Sironi, L. 2021 Secondary energization in compressing plasmoids during magnetic reconnection. Astrophys. J. 912, 48.

[ref35] Hoshino, M. , Mukai, T. , Terasawa, T. & Shinohara, I. 2001 Suprathermal electron acceleration in magnetic reconnection. J. Geophys. Res. 106, 25979.

[ref36] Jaroschek, C.H. , Lesch, H. & Treumann, R.A. 2004 Relativistic kinetic reconnection as the possible source mechanism for high variability and flat spectra in extragalactic radio sources. ApJL 605, L9–L12.

[ref37] Kilian, P. , Li, X. , Guo, F. & Zhang, Q. 2020 Exploring the acceleration mechanisms for particle injection and power-law formation during transrelativistic magnetic reconnection. ApJ 899, 15.

[ref38] Larrabee, D.A. , Lovelace, R.V.E. & Romanova, M.M. 2003 Lepton acceleration by relativistic collisionless magnetic reconnection. Astrophys. J. 586, 72–78.

[ref39] Lemoine, M. 2019 Generalized fermi acceleration. Phys. Rev. *D* 99, 083006.

[ref40] Lemoine, M. & Malkov, M.A. 2020 Power-law spectra from stochastic acceleration. Mon. Not. R. Astron. Soc. 499, 4972–4983.

[ref42] Li, X. , Guo, F. , Li, H. & Li, S. 2018 Large-scale compression acceleration during magnetic reconnection in a low- 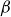 plasma. ApJ 866, 4.

[ref41] Li, X. , Guo, F. & Li, H. 2019 *a* P*a*rticle acceleration in kinetic simulations of nonrelativistic magnetic reconnection with different ion–electron mass ratios. Astrophys. J. 879, 12.

[ref43] Li, X. , Guo, F. , Li, H. , Stanier, A. & Kilian, P. 2019 *b* Formation of power-law electron energy spectra in three-dimensional low- 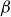 magnetic reconnection. Astrophys. J. 884, 118.

[ref44] Li, X. , Guo, F. , Liu, Y.-H. & Li, H. 2023 A model for nonthermal particle acceleration in relativistic magnetic reconnection. Astrophys. J. Lett. 954, L37.

[ref45] Liu, Y.-H. , Guo, F. , Daughton, W. , Li, H. & Hesse, M. 2015 Scaling of magnetic reconnection in relativistic collisionless pair plasmas. Phys. Rev. Lett. 114, 095002. 25793820 10.1103/PhysRevLett.114.095002

[ref46] Liu, Y.-H. , Hesse, M. , Guo, F. , Daughton, W. , Li, H. , Cassak, P.A. & Shay, M.A. 2017 Why does steady-state magnetic reconnection have a maximum local rate of order 0.1? Phys. Rev. Lett. 118, 085101. 28282209 10.1103/PhysRevLett.118.085101

[ref47] Liu, Y.-H. , Lin, S.-C. , Hesse, M. , Guo, F. , Li, X. , Zhang, H. & Peery, S. 2020 The critical role of collisionless plasma energization on the structure of relativistic magnetic reconnection. ApJL 892, L13.

[ref48] Majeski, S. & Ji, H. 2023 Super-fermi acceleration in multiscale MHD reconnection. Phys. Plasmas 30, 042106.

[ref49] Mehlhaff, J.M. , Werner, G.R. , Uzdensky, D.A. & Begelman, M.C. 2020 Kinetic beaming in radiative relativistic magnetic reconnection: a mechanism for rapid gamma-ray flares in jets. Mon. Not. R. Astron. Soc. 498, 799–820.

[ref50] Mehlhaff, J.M. , Zhou, M. & Zhdankin, V. 2025 Radiative relativistic turbulence as an in situ pair-plasma source in blazar jets. Astrophys. J. 987, 159.

[ref51] Melzani, M. , Walder, R. , Folini, D. , Winisdoerffer, C. & Favre, J.M. 2014 Relativistic magnetic reconnection in collisionless ion-electron plasmas explored with particle-in-cell simulations. Astron. Astrophys. 570, A111.

[ref52] Nalewajko, K. , Uzdensky, D.A. , Cerutti, B. , Werner, G.R. & Begelman, M.C. 2015 On the distribution of particle acceleration sites in plasmoid-dominated relativistic magnetic reconnection. Astrophys. J. 815, 101.

[ref53] Parsons, J. , Spitkovsky, A. & Vanthieghem, A. 2024 Microphysics of particle reflection in weibel-mediated shocks. Astrophys. J. 971, 18.

[ref54] Petropoulou, M. & Sironi, L. 2018 The steady growth of the high-energy spectral cut-off in relativistic magnetic reconnection. MNRAS 481, 5687–5701.

[ref55] Rowan, M.E. , Sironi, L. & Narayan, R. 2017 Electron and proton heating in transrelativistic magnetic reconnection. Astrophys. J. 850, 29.

[ref56] Schoeffler, K.M. , Grismayer, T. , Uzdensky, D. , Fonseca, R.A. & Silva, L.O. 2019 Bright gamma-ray flares powered by magnetic reconnection in QED-strength magnetic fields. Astrophys. J. 870, 49.

[ref57] Sironi, L. 2022 Nonideal fields solve the injection problem in relativistic reconnection. Phys. Rev. Lett. 128, 145102. 35476488 10.1103/PhysRevLett.128.145102

[ref58] Sironi, L. & Beloborodov, A.M. 2020 Kinetic simulations of radiative magnetic reconnection in the coronae of accreting black holes. Astrophys. J. 899, 52.

[ref59] Sironi, L. & Spitkovsky, A. 2011 Acceleration of particles at the termination shock of a relativistic striped wind. ApJ 741, 39.

[ref60] Sironi, L. & Spitkovsky, A. 2014 Relativistic reconnection: an efficient source of non-thermal particles. Astrophys. J. 783, L21.

[ref61] Sironi, L. , Uzdensky, D.A. & Giannios, D. 2025 Relativistic magnetic reconnection in astrophysical plasmas: a powerful mechanism of nonthermal emission. Annu. Rev. Astron. Astr. 63, 127–178.

[ref62] Speiser, T.W. 1965 Particle trajectories in model current sheets: 1. Analytical solutions. J. Geophys. Res. 70, 4219–4226.

[ref63] Spitkovsky, A. 2008 Particle acceleration in relativistic collisionless shocks: Fermi process at last? Astrophys. J. 682, L5–L8.

[ref64] Totorica, S.R. , Zenitani, S. , Matsukiyo, S. , Machida, M. , Sekiguchi, K. & Bhattacharjee, A. 2023 Exact calculation of nonideal fields demonstrates their dominance of injection in relativistic reconnection. ApJL 952, L1.

[ref65] Uzdensky, D.A. 2022 Relativistic non-thermal particle acceleration in two-dimensional collisionless magnetic reconnection. J. Plasma Phys. 88, 905880114. 35241860 10.1017/S0022377822000046 PMC8886498

[ref66] Uzdensky, D.A. , Cerutti, B. & Begelman, M.C. 2011 Reconnection-powered linear accelerator and gamma-ray flares in the crab nebula. Astrophys. J. 737, L40.

[ref67] Werner, G.R. & Uzdensky, D.A. 2017 Nonthermal particle acceleration in 3d relativistic magnetic reconnection in pair plasma. Astrophys. J. Lett. 843, L27. 10.1017/S0022377822000046 PMC8886498 35241860

[ref68] Werner, G.R. & Uzdensky, D.A. 2021 Reconnection and particle acceleration in three-dimensional current sheet evolution in moderately magnetized astrophysical pair plasma. J. Plasma Phys. 87, 905870613.

[ref69] Werner, G.R. , Uzdensky, D.A. , Begelman, M.C. , Cerutti, B. & Nalewajko, K. 2018 Non-thermal particle acceleration in collisionless relativistic electron–proton reconnection. Mon. Not. R. Astron. Soc. 473, 4840–4861.

[ref70] Werner, G.R. , Uzdensky, D.A. , Cerutti, B. , Nalewajko, K. & Begelman, M.C. 2016 The extent of power-law energy spectra in collisionless relativistic magnetic reconnection in pair plasmas. Astrophys. J. Lett. 816, L8.

[ref71] Wong, K. , Zhdankin, V. , Uzdensky, D.A. , Werner, G.R. & Begelman, M.C. 2020 First-principles demonstration of diffusive-advective particle acceleration in kinetic simulations of relativistic plasma turbulence. Astrophys. J. 893, L7.

[ref72] Zenitani, S. & Hoshino, M. 2001 The generation of nonthermal particles in the relativistic magnetic reconnection of pair plasmas. Astrophys. J. 562, L63–L66.

[ref73] Zenitani, S. & Hoshino, M. 2005 Three-dimensional evolution of a relativistic current sheet: triggering of magnetic reconnection by the guide field. Phys. Rev. Lett. 95, 09500. 10.1103/PhysRevLett.95.095001 16197219

[ref74] Zenitani, S. & Hoshino, M. 2008 The role of the guide field in relativistic pair plasma reconnection. Astrophys. J. 677, 530–544.

[ref75] Zhang, H. , Sironi, L. & Giannios, D. 2021 Fast particle acceleration in three-dimensional relativistic reconnection. ApJ 922, 261.

[ref76] Zhang, H. , Sironi, L. , Giannios, D. & Petropoulou, M. 2023 The origin of power-law spectra in relativistic magnetic reconnection. Astrophys. J. Lett. 956, L36.

[ref77] Zhang, Q. , Drake, J.F. & Swisdak, M. 2019 Particle heating and energy partition in low- 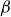 guide field reconnection with kinetic Riemann simulations. Phys. Plasmas 26, 072115.

[ref78] Zhang, Q. , Guo, F. , Daughton, W. , Li, H. , Le, A. , Phan, T. & Desai, M. 2024 Multispecies ion acceleration in 3d magnetic reconnection with hybrid-kinetic simulations. Phys. Rev. Lett. 132, 115201. 38563953 10.1103/PhysRevLett.132.115201

[ref79] Zhang, Q. , Guo, F. , Daughton, W. , Li, H. & Li, X. 2021 Efficient nonthermal ion and electron acceleration enabled by the flux-rope kink instability in 3d nonrelativistic magnetic reconnection. Phys. Rev. Lett. 127, 185101. 34767407 10.1103/PhysRevLett.127.185101

[ref80] Zhdankin, V. , Uzdensky, D.A. , Werner, G.R. & Begelman, M.C. 2018 System-size convergence of nonthermal particle acceleration in relativistic plasma turbulence. Astrophys. J. Lett. 867, L18.

[ref81] Zhdankin, V. , Uzdensky, D.A. , Werner, G.R. & Begelman, M.C. 2019 Electron and ion energization in relativistic plasma turbulence. Phys. Rev. Lett. 122, 055101. 30822031 10.1103/PhysRevLett.122.055101

[ref82] Zhdankin, V. , Werner, G.R. , Uzdensky, D.A. & Begelman, M.C. 2017 Kinetic turbulence in relativistic plasma: from thermal bath to nonthermal continuum. Phys. Rev. Lett. 118, 055103. 28211730 10.1103/PhysRevLett.118.055103

